# Supplementary material for: O-GlcNAcylation enhances CPS1 catalytic efficiency for ammonia and promotes ureagenesis
Source: Nat Commun. 2022 Sep 5;13:5212. doi: 10.1038/s41467-022-32904-x (PMC9445089; doi:10.1038/s41467-022-32904-x)
Supplement: Supplementary file 1 — Supplementary Information [file 41467_2022_32904_MOESM1_ESM.pdf]

Supplementary Information

O-GlcNAcylation enhances CPS1 catalytic efficiency for ammonia and promotes ureagenesis

Leandro R. Soria, Georgios Makris, Alfonso M. D'Alessio, Angela De Angelis, Iolanda Boffa, Veronica M. Pravata, Véronique Rüfenacht, Sergio Attanasio, Edoardo Nusco, Paola Arena, Andrew T. Ferenbach, Debora Paris, Paola Cuomo, Andrea Motta, Matthew Nitzahn, Gerald S. Lipshutz, Ainhoa Martínez-Pizarro, Eva Richard, Lourdes R. Desviat, Johannes Häberle, Daan M.F. van Aalten, and Nicola Brunetti-Pierri.

Correspondence to: l.soria@tigem.it; brunetti@tigem.it

SUPPLEMENTARY FIGURES

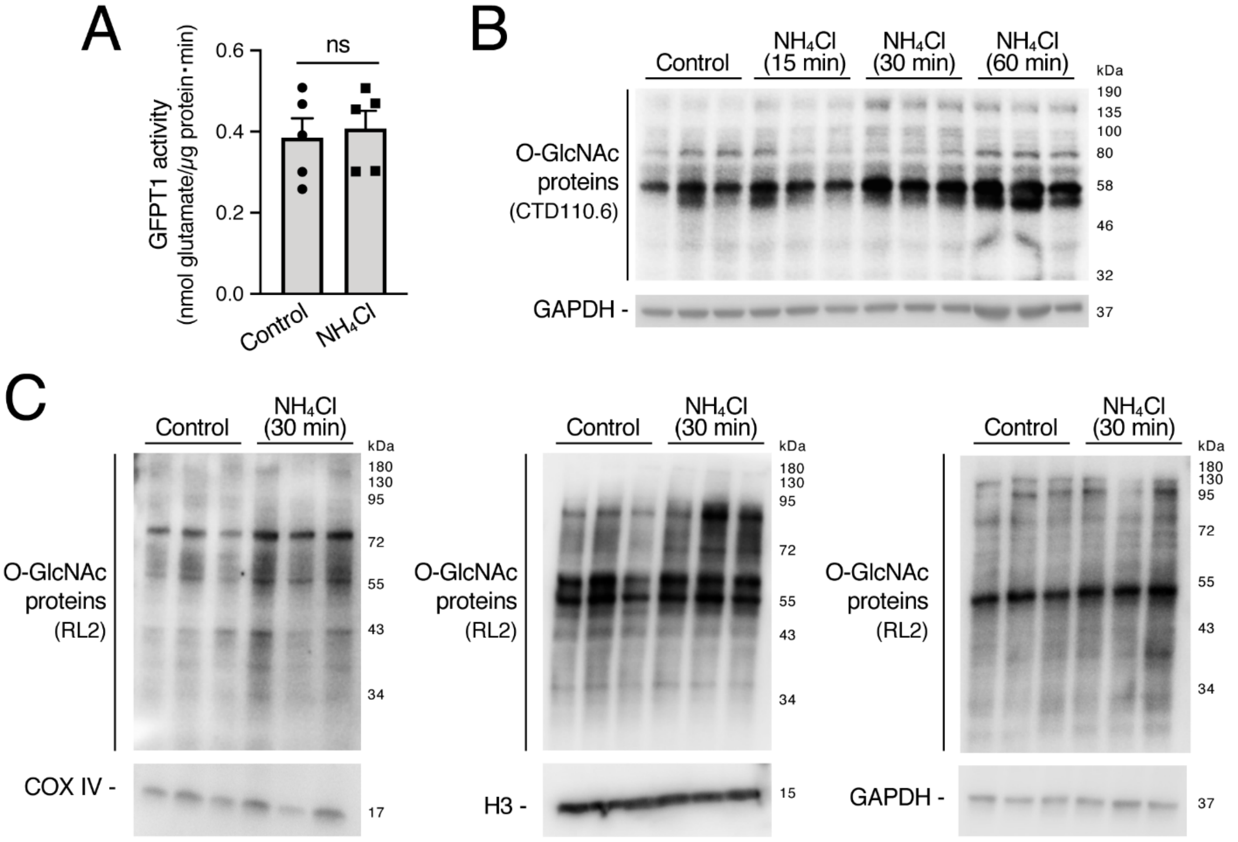

Supplementary Fig. 1. Hyperammonemia-induced hepatic O-GlcNAcylation. (A) Liver GFPT1 catalytic activity in livers of C57BL/6 wild-type (WT) mice harvested 1.0 hour after the intraperitoneal (i.p.) injection of ammonium chloride (NH_4Cl) (10 mmol/kg) compared to livers of mice that received i.p. injection of sodium chloride (Control) (n=5 mice/group). (**B)** Western blot for O-GlcNAc proteins with the CTD110.6 antibody on livers of WT mice harvested at various times (15, 30, and 60 min) after the i.p. injection of NH_4Cl (10 mmol/kg) or sodium chloride as control (Control) (n=3 mice/group). GAPDH was used as loading control. **(C)** Western blot for O-GlcNAc proteins with the RL2 antibody on mitochondrial, nuclear and cytoplasmic subcellular fractions from livers of WT mice harvested 30 min after the i.p. injection of NH_4Cl (10 mmol/kg) or sodium chloride as control (Control) (n=3 mice/group). COX IV, H3, and GAPDH were used as loading controls and to confirm fraction enrichment. Values are shown as averages ± S.E.M. Abbreviations: ns, no statistically significant difference. Experiments in panels **B** and **C** were performed twice.

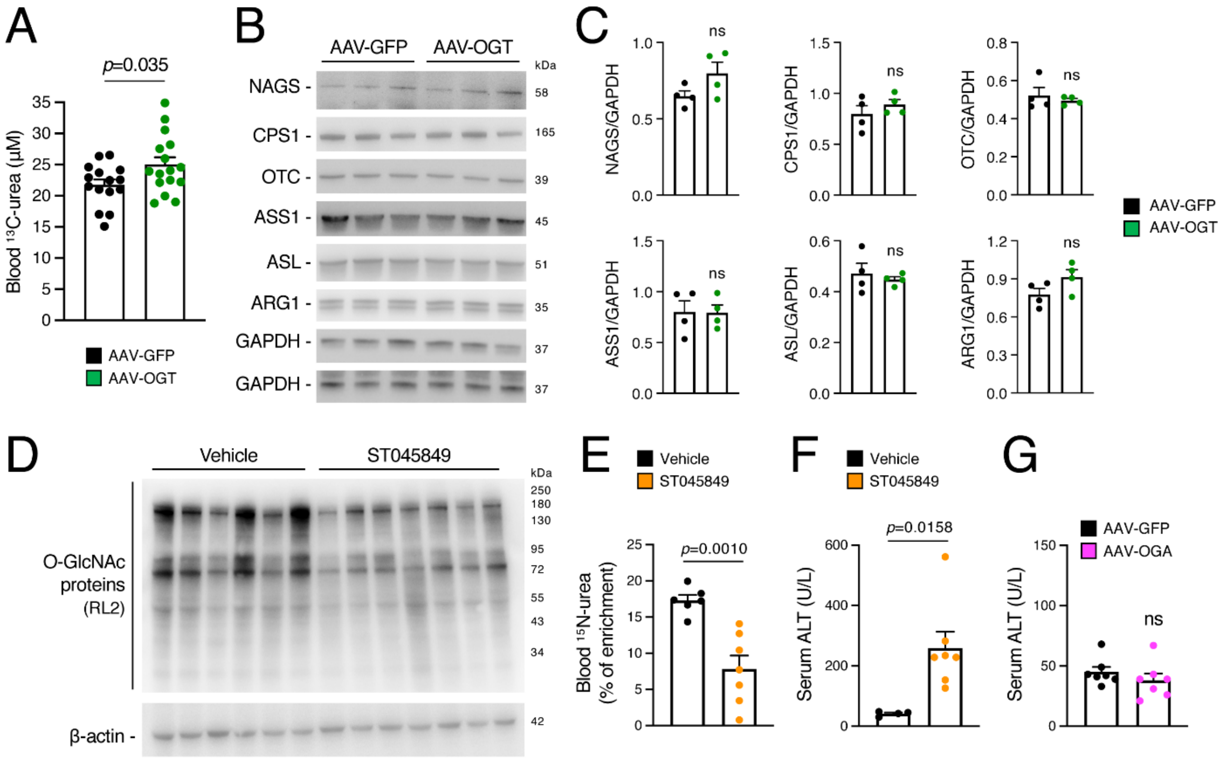

Supplementary Fig. 2. Expression of urea cycle enzymes in livers of mice overexpressing OGT and ureagenesis in mice treated with an OGT inhibitor. (A) Blood ^{13}C-urea measured 30 min after administration of sodium acetate-1-^{13}C (55 mg/kg, i.p.) in AAV-OGT- (n=16) and AAV-GFP-injected (n=15) C57BL/6 wild-type (WT) mice. *p*=0.035 (Unpaired t-test). **(B)** Western blots of urea cycle enzymes (NAGS, CPS1, OTC, ASS1, ASL, and ARG1) in livers of WT mice harvested four weeks after the intravenous (i.v.) injections of AAV-OGT or AAV-GFP. GAPDH was used as loading control and **(C)** corresponding densitometric quantifications (n=4 mice/group). **(D)** Western blot of O-GlcNAc proteins with RL2 antibody in livers harvested 30 min after the i.p. injection of NH_4Cl (10 mmol/kg) of WT mice that were injected i.p. 18 hours and 2 hours before with an OGT inhibitor (ST045849, 40 mg/kg). β-actin was used as loading control. **(E)** Isotopic enrichment of ^{15}N-urea in blood 30 min after i.p. injection of $^{15}NH_4Cl$ (10 mmol/kg) in WT mice injected with ST045849 (n=7) or vehicle (n=6). *p*=0.0010 (Unpaired t-test). **(F)** Serum alanine transaminase (ALT) levels in WT mice injected with ST045849 (n=7) or vehicle (n=4). *p*=0.0158 (Unpaired t-test). **(G)** Serum ALT levels in WT mice injected with AAV-OGA or AAV-GFP (n=7 mice/group). All values are shown as averages ± S.E.M. Abbreviations: ns, no statistically significant difference.

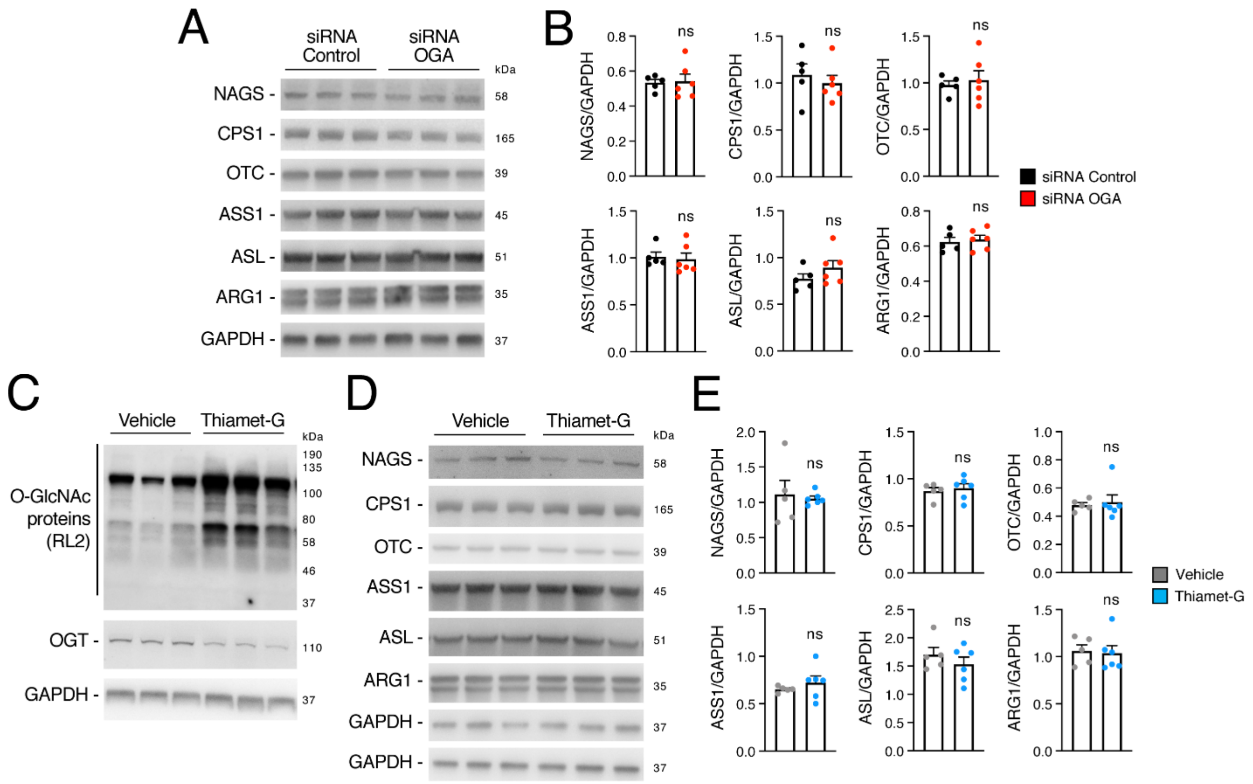

Supplementary Fig. 3. Expression of urea cycle enzymes in mice with hepatic downregulation of OGA. (A) Western blots for urea cycle enzymes (NAGS, CPS1, OTC, ASS1, ASL, and ARG1) in livers of C57BL/6 wild-type (WT) mice 6 days after i.v. administration of a mix of control siRNA (1 mg/kg) or a siRNA against mouse OGA mRNA. GAPDH was used as loading control and **(B)** corresponding densitometric quantifications (siRNA control, n=5; siRNA OGA, n=6). **(C)** Western blot for O-GlcNAc proteins (RL2 antibody) and OGT in livers of WT mice treated with Thiamet-G (40 mg/kg, i.p. for 2 days) or vehicle (PBS). GAPDH was used as loading control. **(D)** Western blot analyses of urea cycle enzymes in livers of WT mice injected with Thiamet-G (40 mg/kg i.p. for two days) or vehicle (PBS) as controls. GAPDH was used as loading control **(E)** and corresponding densitometric quantifications (Vehicle, n=5; Thiamet-G, n=6). All values are shown as averages ± S.E.M. Abbreviations: ns, no statistically significant difference.

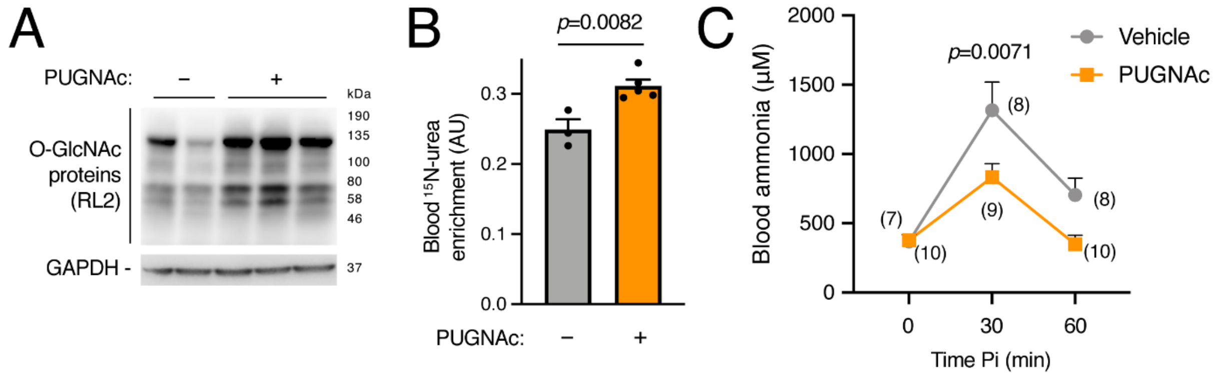

Supplementary Fig. 4. Increased ureagenesis and ammonia detoxification in mice treated with PUGNAc. (A) Western blot of O-GlcNAc proteins (RL2 antibody) in livers of C57BL/6 wild-type (WT) mice treated with PUGNAc (7 mg/kg i.p. for 2 days) or vehicle (PBS) as controls. GAPDH was used as loading control. **(B)** Blood levels of ^{15}N-urea 30 min after the i.p. injection of $^{15}NH_4Cl$ (10 mmol/kg) in mice pre-treated with vehicle (n=3) or PUGNAc-treated mice (n=5). *p*=0.0082 (Unpaired t-test). **(C)** Blood ammonia at baseline, 30 and 60 min after i.p. injection of $^{15}NH_4Cl$ (10 mmol/kg) in WT mice treated with vehicle or PUGNAc. The number between parentheses indicates the number of mice for each time-point. *p*=0.0071 (Two-way ANOVA). All values are shown as averages ± S.E.M. Abbreviations: AU, arbitrary units; Pi, post-injection.

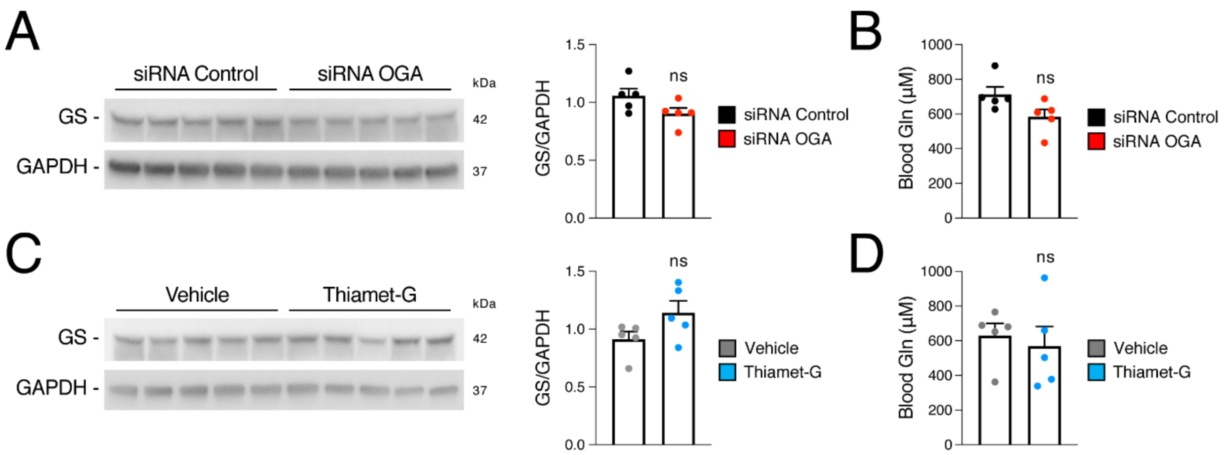

Supplementary Fig. 5. Glutamine synthetase (GS) expression and blood glutamine concentrations in mice with enhanced hepatic O-GlcNAcylation. (A) Western blots and densitometric quantification of GS in livers of C57BL/6 wild-type (WT) mice with siRNA-induced OGA downregulation (1 mg/kg i.v. 6 days prior to sacrifice, n=5 mice/group). **(B)** Blood glutamine concentrations at 30 min after i.p. injections of NH_4Cl (10 mmol/kg) in control siRNA and OGA-siRNA-injected mice (n=5 mice/group). **(C)** Western blot bands and densitometric quantification of GS in livers of WT mice treated with Thiamet-G (40 mg/kg i.p. for two days) or vehicle (n=5 mice/group). GAPDH was used as loading control. **(D)** Blood glutamine 30 min after i.p. injections of NH_4Cl (10 mmol/kg) in Thiamet-G- or vehicle-treated mice (n=5 mice/group). All values are shown as averages ± S.E.M. Abbreviations: Gln, glutamine; ns, no statistically significant difference.

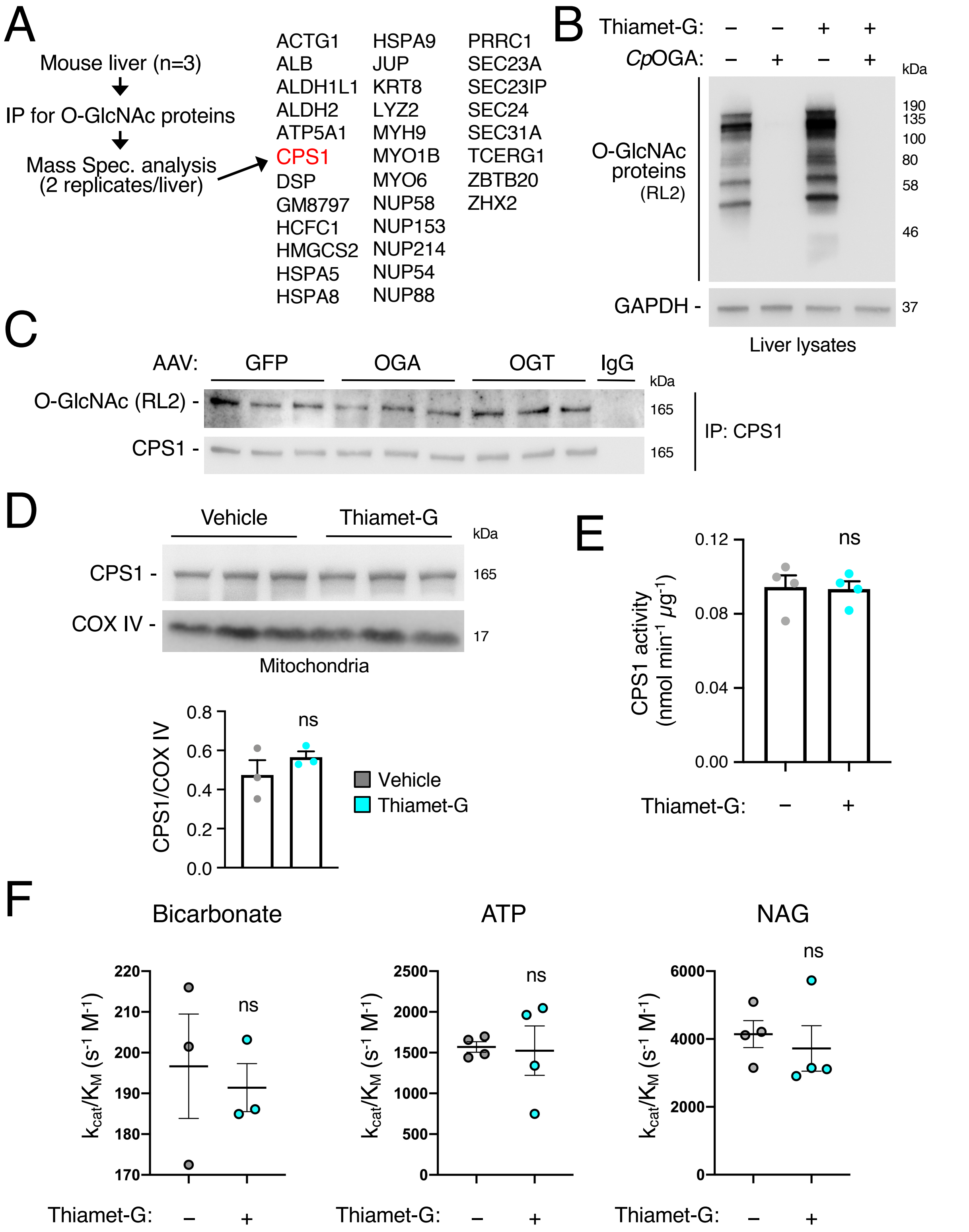

Supplementary Fig. 6. O-GlcNAcylation of CPS1 and effect of Thiamet-G on CPS1 function. **(A)** Proteins identified by MS on liver lysates immunoprecipitated with an antibody against O-

GlcNAcylated proteins (RL2). Listed proteins were identified in all six independent replicates. Full list of proteins is provided in **Supplementary Data 1**. **(B)** Western blot of O-GlcNAcylated proteins in livers of C57BL/6 wild-type (WT) mice i.p. injected with vehicle (PBS) or Thiamet-G (40 mg/kg for 2 days). Pre-incubation of liver lysates with the bacterial OGA orthologue from *Clostridium perfringens* (*Cp*OGA) that specifically removes the O-GlcNAc moiety resulted in loss of immunoreactivity detected by the anti-O-GlcNAc antibody (RL2). **(C)** Western blot for O-GlcNAcylation (RL2) on CPS1 immunoprecipitates of liver lysates of WT mice injected with AAV vectors expressing GFP, OGA or OGT (n=3 mice/group). Immunoprecipitates with IgG control are also shown. **(D)** Western blot and densitometric quantification of CPS1 in mitochondrial fractions of C57BL/6 wild-type (WT) mice injected with Thiamet-G (40 mg/kg i.p. for two days) or vehicle (PBS) (n=3 mice/group). COX IV was used as loading control. **(E)** CPS1 catalytic activity in livers of WT mice injected with Thiamet-G (40 mg/kg i.p. for two days) or vehicle as controls (n=4 mice/group). **(F)** CPS1 specificity constant (kinetic efficiency) for bicarbonate (n=3 mice/group), ATP (n=4 mice/group), and NAG (n=4 mice/group) in liver tissues of mice treated with Thiamet-G (n=3-4 mice/group). All values are shown as averages ± S.E.M. Abbreviations: IP, immunoprecipitation; NAG, *N*-acetyl-glutamate; ns, no statistically significant difference. Experiments in panel **B** were performed three times, while experiments in panels **C** and **D** were performed once.

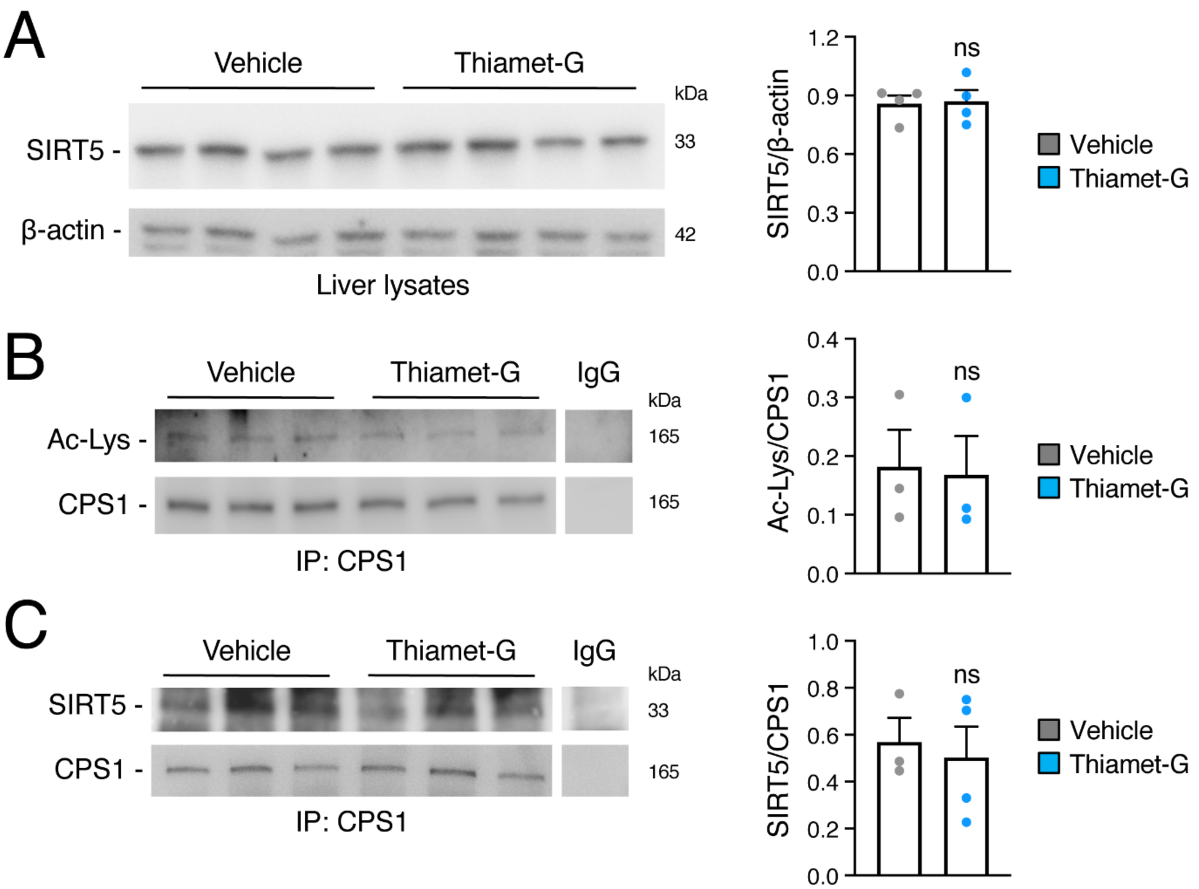

Supplementary Fig. 7. Effect of Thiamet-G on hepatic expression of SIRT5. (A) Western blot and densitometric quantification of sirtuin 5 (SIRT5) in liver lysates from C57BL/6 wild-type (WT) mice injected with Thiamet-G (40 mg/kg i.p. for two days) or vehicle (PBS) as control (n=4 mice/group). β-actin was used as loading control. **(B)** Western blot for acetylation (Ac-Lys) in CPS1 immunoprecipitates of liver lysates of WT mice injected with Thiamet-G (40 mg/kg i.p. for two days) or vehicle (PBS), as control (n=3 mice/group). **(C)** Western blot for SIRT5 on CPS1 immunoprecipitates of liver lysates of WT mice injected with Thiamet-G (40 mg/kg i.p. for two days) (n=4 mice/group) or vehicle (PBS), as control (n=3 mice/group). Immunoprecipitates with IgG control are also shown. All values are shown as averages ± S.E.M. Abbreviations: IP, immunoprecipitation; ns, no statistically significant difference.

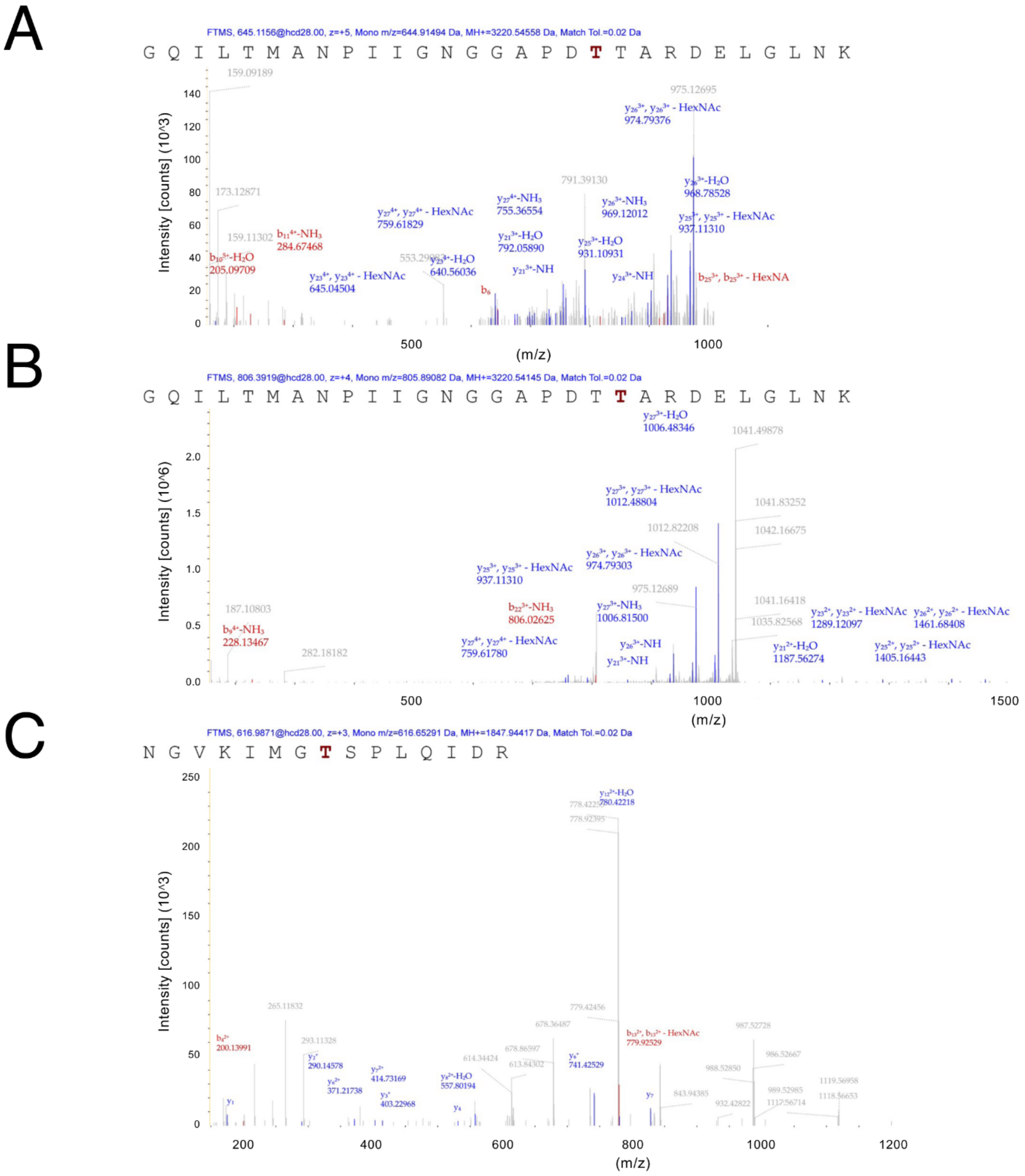

Supplementary Fig. 8. O-GlcNAc site mapping of CPS1 from mouse liver. (A-C) ETD MS spectrum of O-GlcNAcylated CPS1 at Thr109, Thr110 and Thr1078.

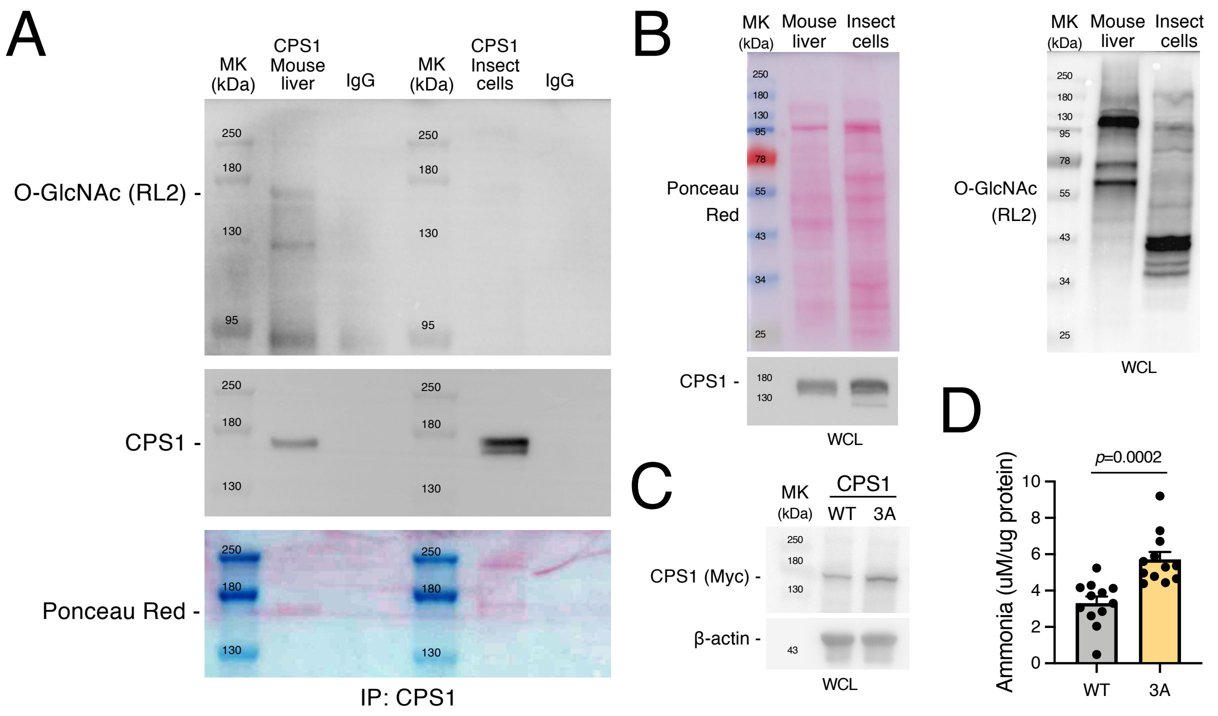

Supplementary Fig. 9. CPS1 overexpression in insect and Huh7 cells. (A) Immunoprecipitated CPS1 is O-GlcNAcylated (RL2 antibody) in livers of C57BL/6 wild-type (WT) mice, but not in insect cells. Immunoprecipitates with IgG control are also shown. **(B)** Western blot of O-GlcNAcylated proteins and CPS1 in mouse livers lysates and insect cell extracts. Ponceau Red staining showed uniform protein loading. **(C)** Western blot for Myc-tagged WT and triple mutant (3A) CPS1 in Huh7. β-actin was used as loading control. **(D)** Huh7 cells expressing either WT or 3A CPS1 were incubated with 1 mM ammonium chloride to evaluate ammonia removal capacity. Ammonia was determined in the culture media after 24 hours of incubation with ammonium (n=12 replicates/group). p=0.0002 (Unpaired t-test). Values are shown as averages ± S.E.M. Abbreviations: IP, immunoprecipitation; WCL, whole cell lysate. Experiments in panels **A** and **B** were performed twice.

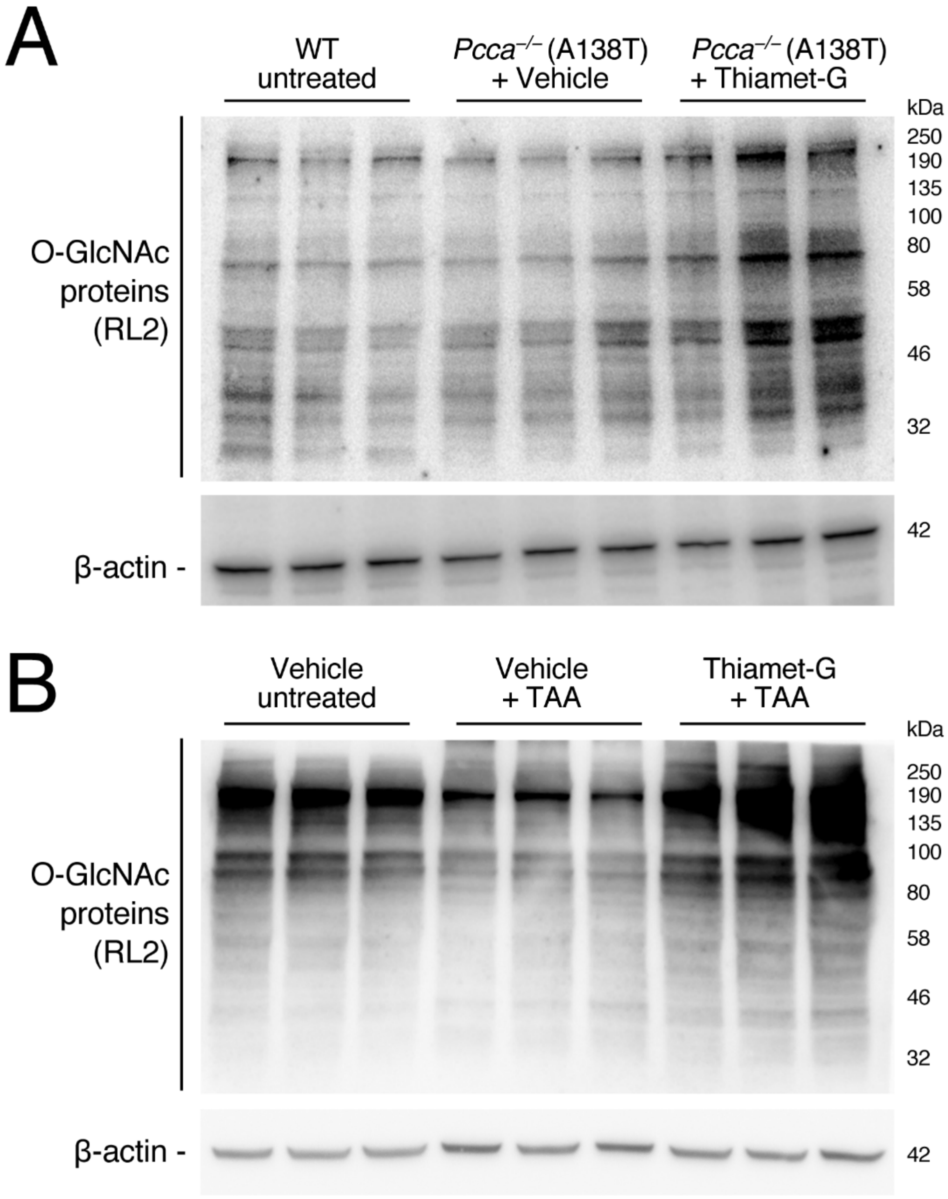

Supplementary Fig. 10. Protein O-GlcNAcylation in an inherited and an acquired liver disease model after treatment with Thiamet-G. (A) Western blot of O-GlcNAc proteins (RL2 antibody) in livers of *Pcca*$^{-/-}$ (A138T) mice injected with Thiamet-G (40 mg/kg for 5 days) or vehicle (PBS). Age-matched C57BL/6 wild-type (WT) untreated mice were used as controls. (n=3 mice/group). β-actin was used as loading control. **(B)** Western blot for O-GlcNAc proteins with the RL2 antibody on livers of WT mice treated with Thiamet-G (40 mg/kg, i.p. for 5 days) and/or thioacetamide (TAA; 250 mg/kg, i.p. 24 hours before sacrifice) or with vehicle. (n=3 mice/group). β-actin was used as loading control.

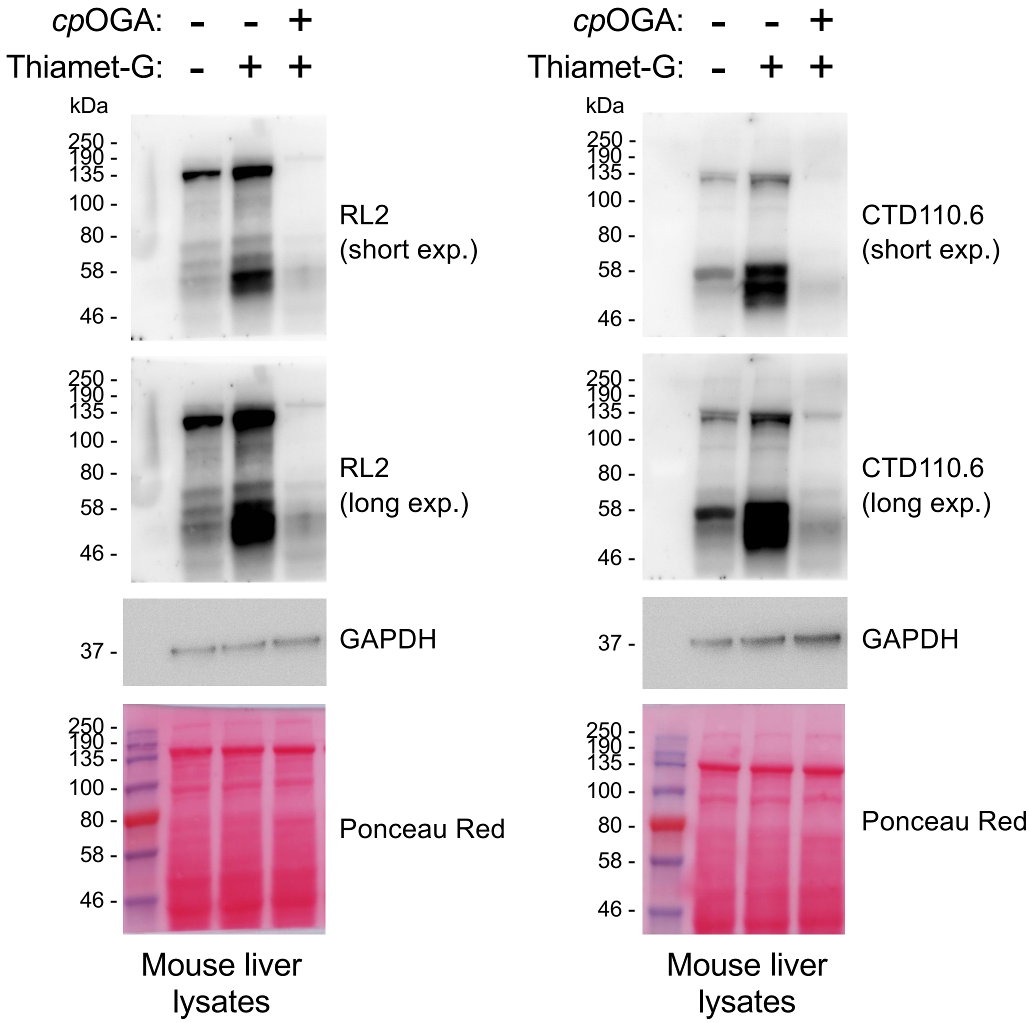

Supplementary Fig. 11. Validation of anti-O-GlcNAc antibodies. Western blot of O-GlcNAcylated proteins with the RL2 and CTD110.6 antibodies in livers of C57BL/6 wild-type (WT) mice injected intraperitoneally with vehicle (PBS) or Thiamet-G (40 mg/kg for 2 days). The signal is specific to O-GlcNAc–modified proteins, as pre-incubation of liver lysates with the bacterial OGA orthologue from *Clostridium perfringens* (*Cp*OGA), that specifically removes the O-GlcNAc moiety, resulted in loss immunoreactivity. GAPDH and Ponceau Red staining are showed as loading controls.

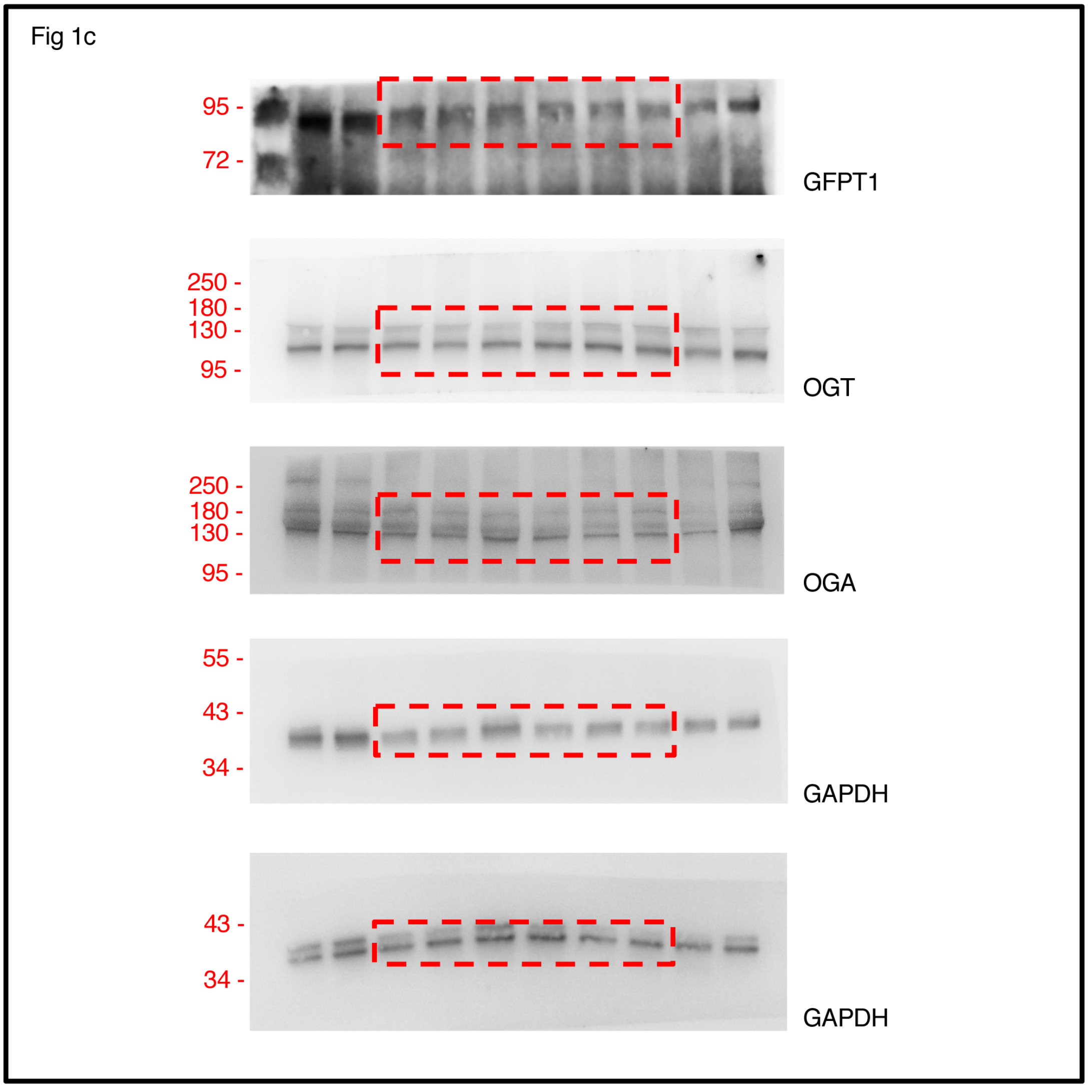

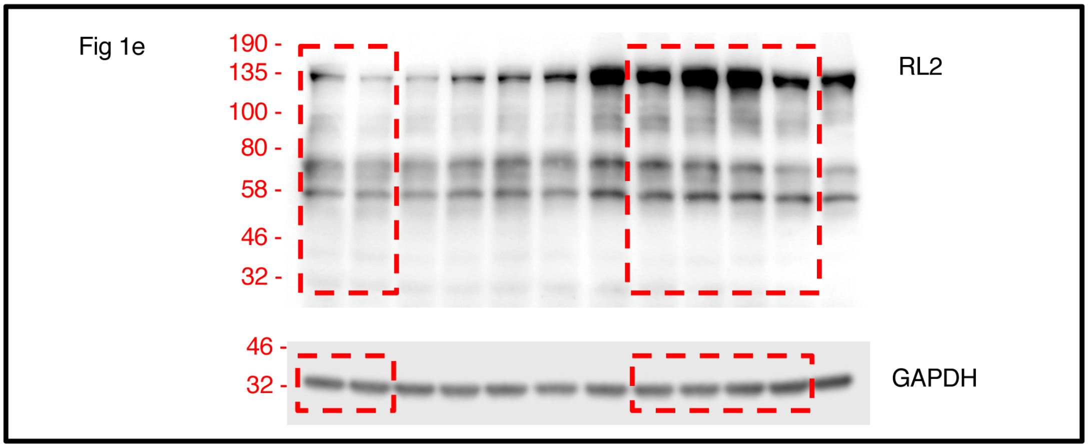

Supplementary Fig. 12. Uncropped blot scans and microscopy images. Part 1.

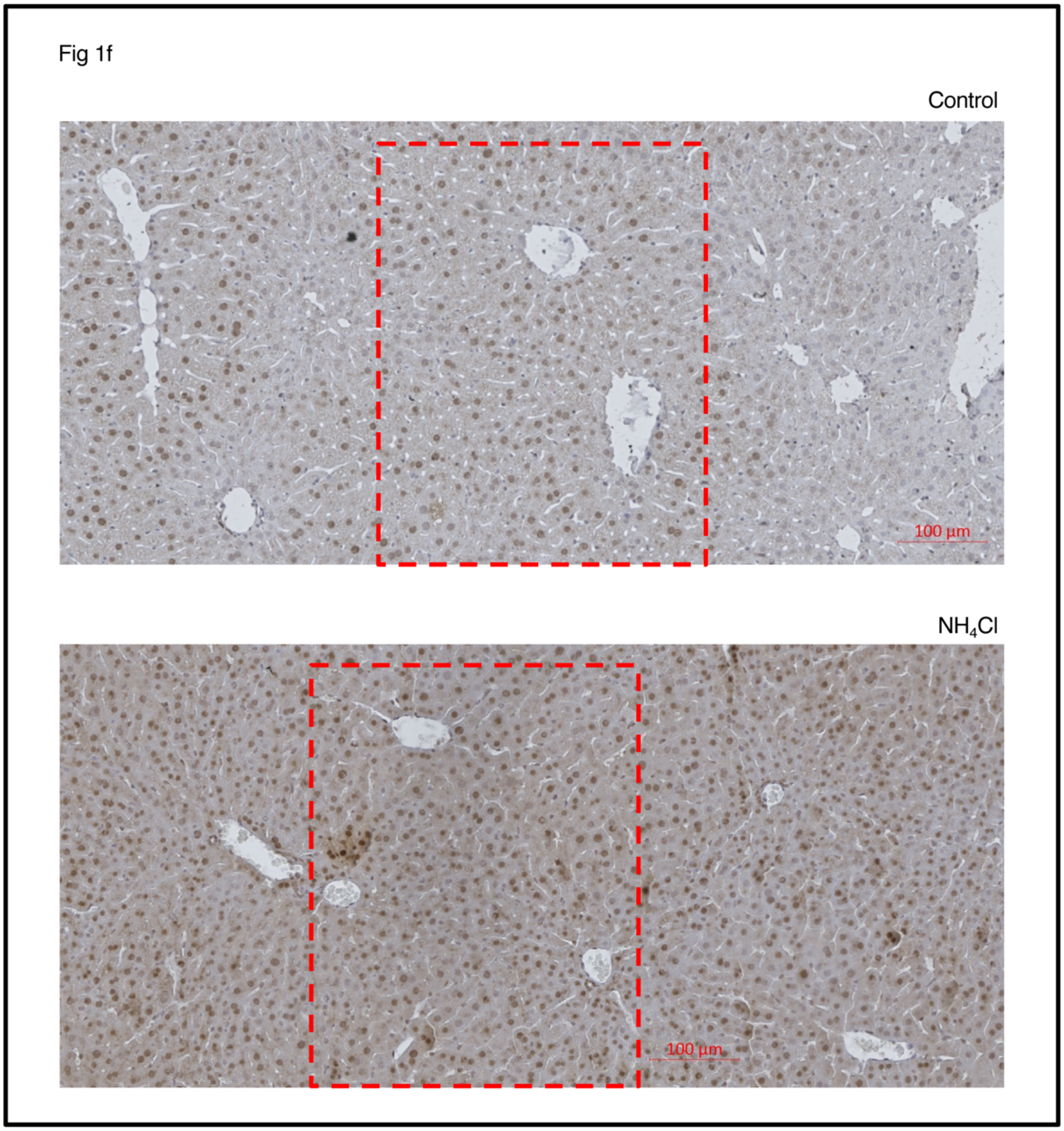

Supplementary Fig. 12. Uncropped blot scans and microscopy images. Part 2.

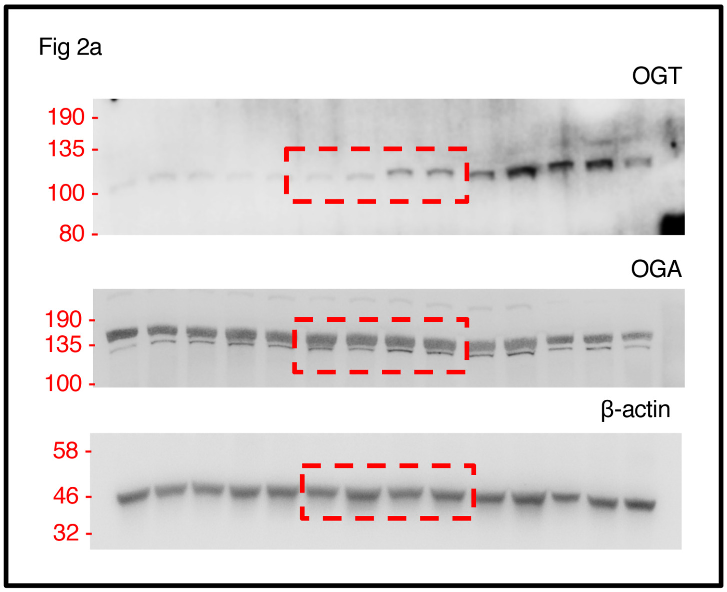

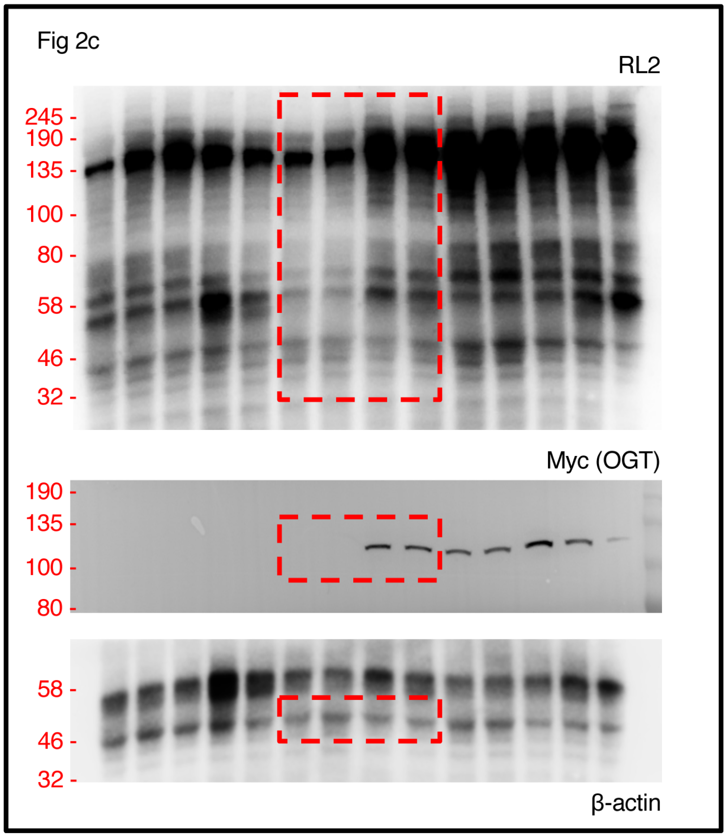

Supplementary Fig. 12. Uncropped blot scans and microscopy images. Part 3.

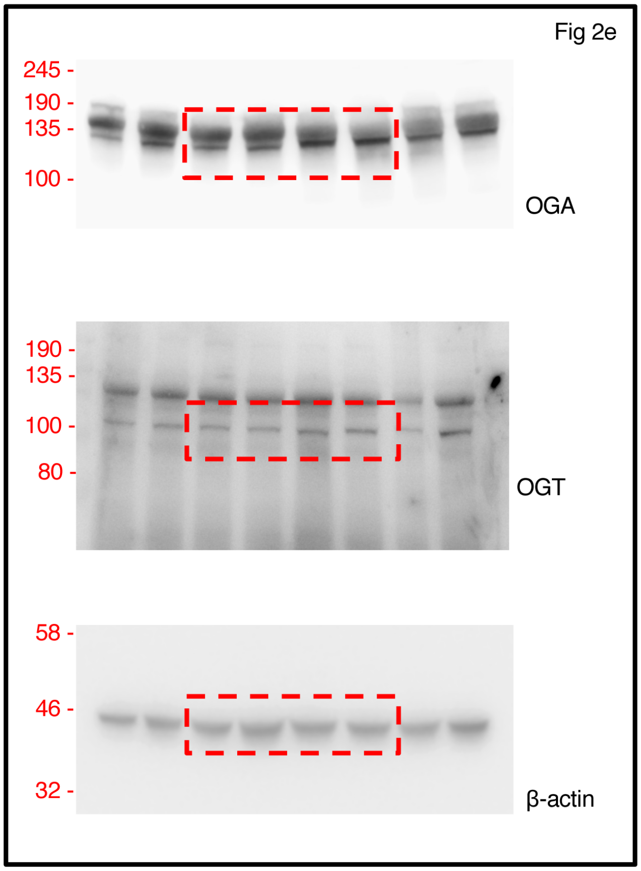

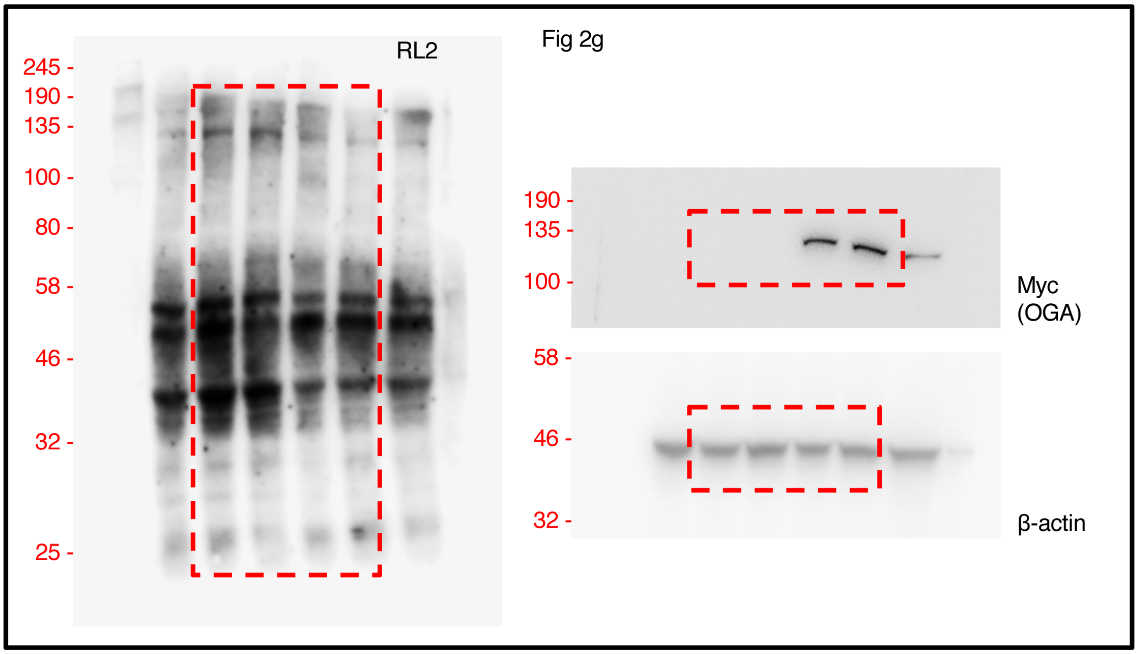

Supplementary Fig. 12. Uncropped blot scans and microscopy images. Part 4.

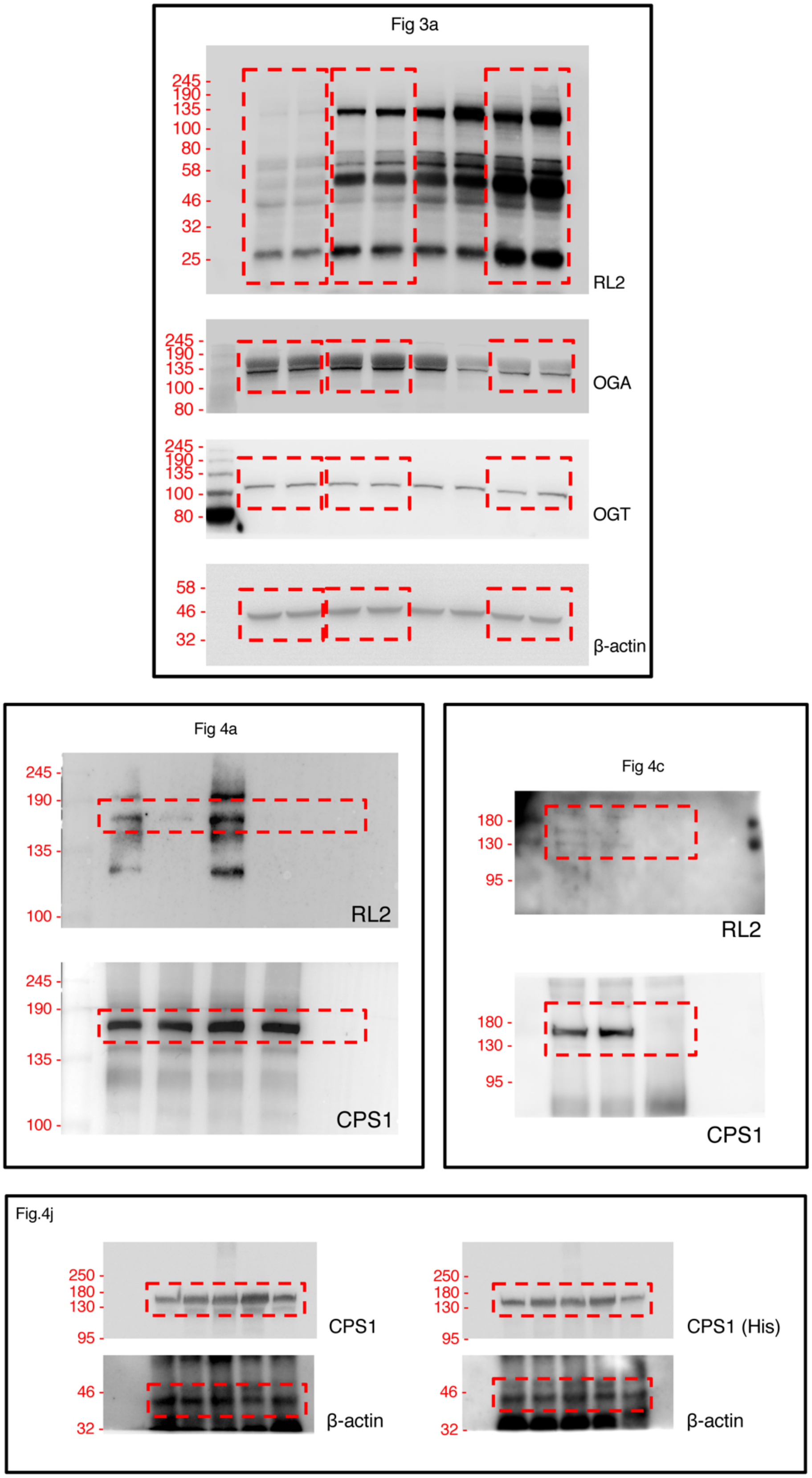

Supplementary Fig. 12. Uncropped blot scans and microscopy images. Part 5.

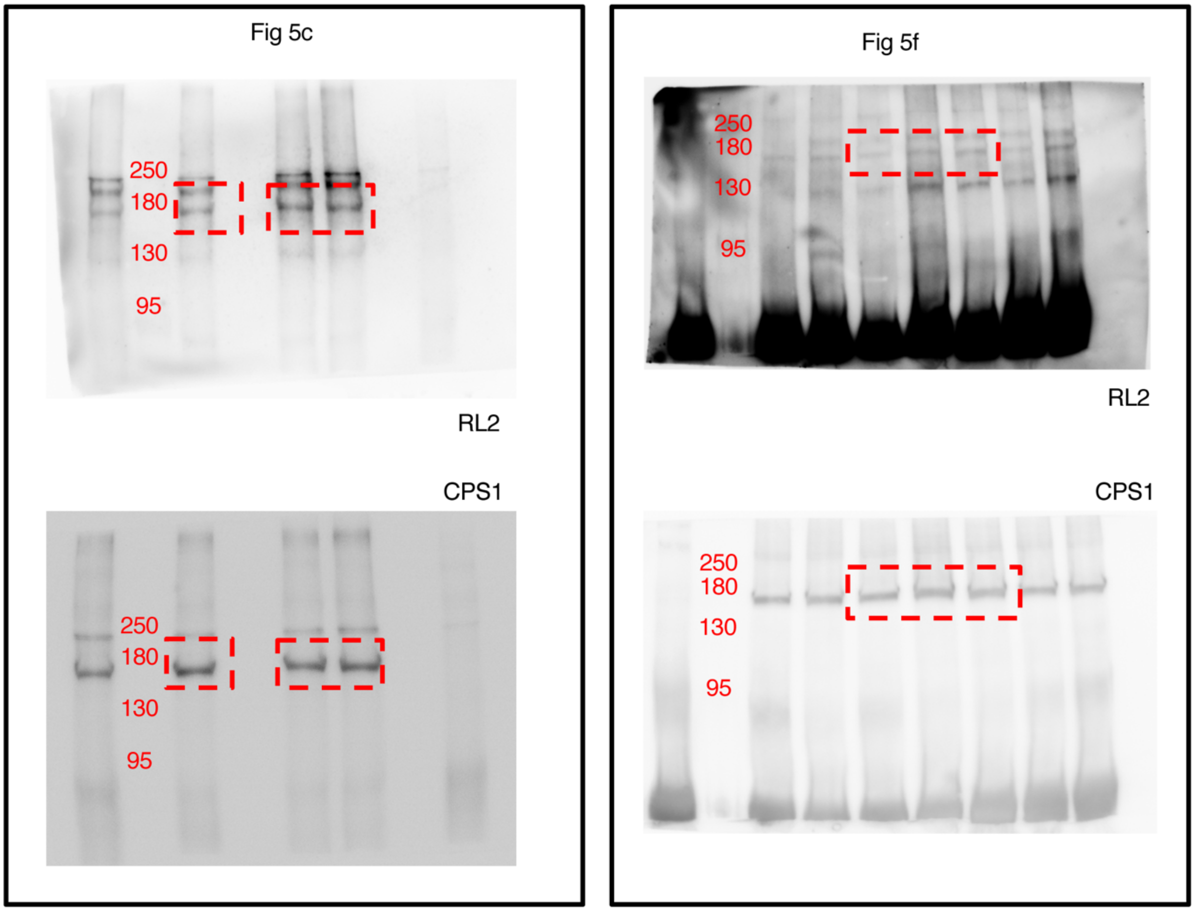

Supplementary Fig. 12. Uncropped blot scans and microscopy images. Part 6.

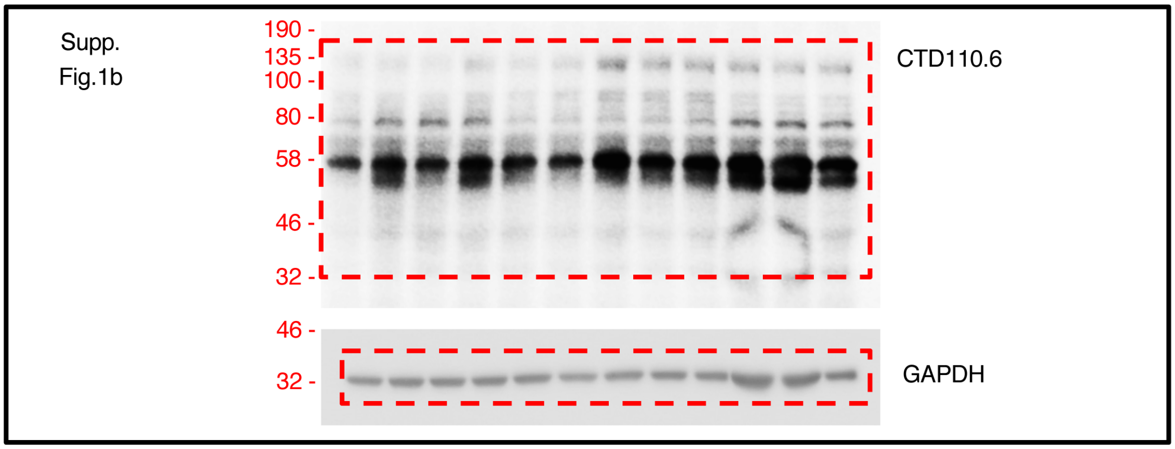

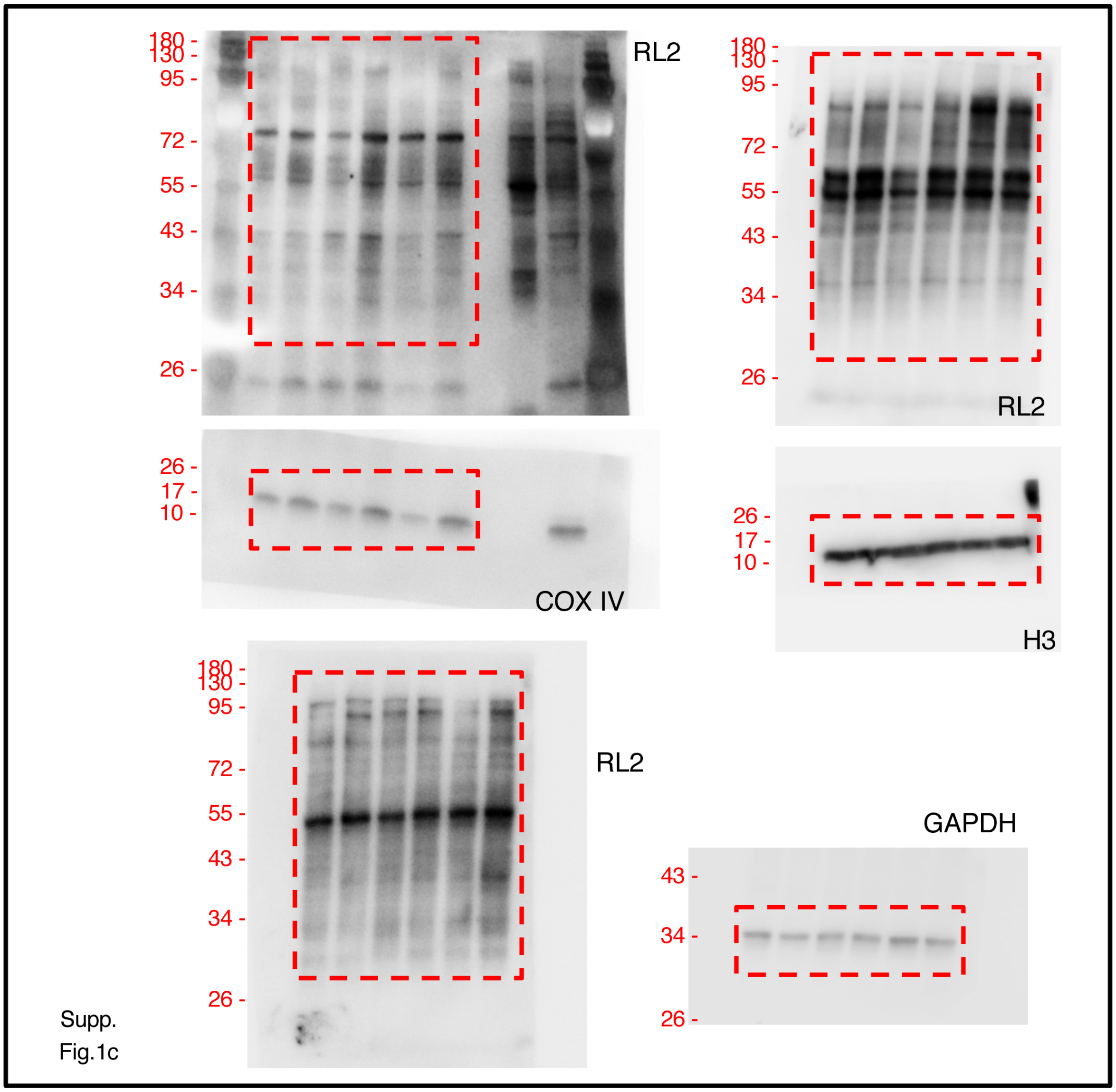

Supplementary Fig. 12. Uncropped blot scans and microscopy images. Part 7.

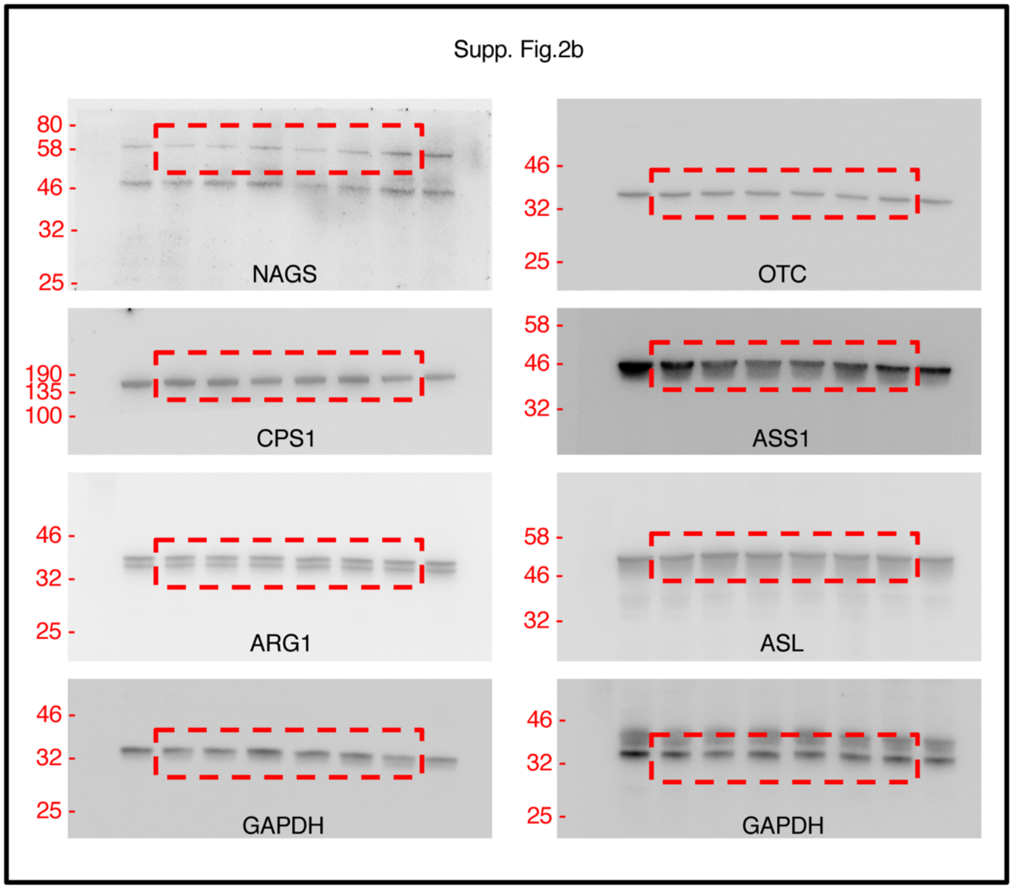

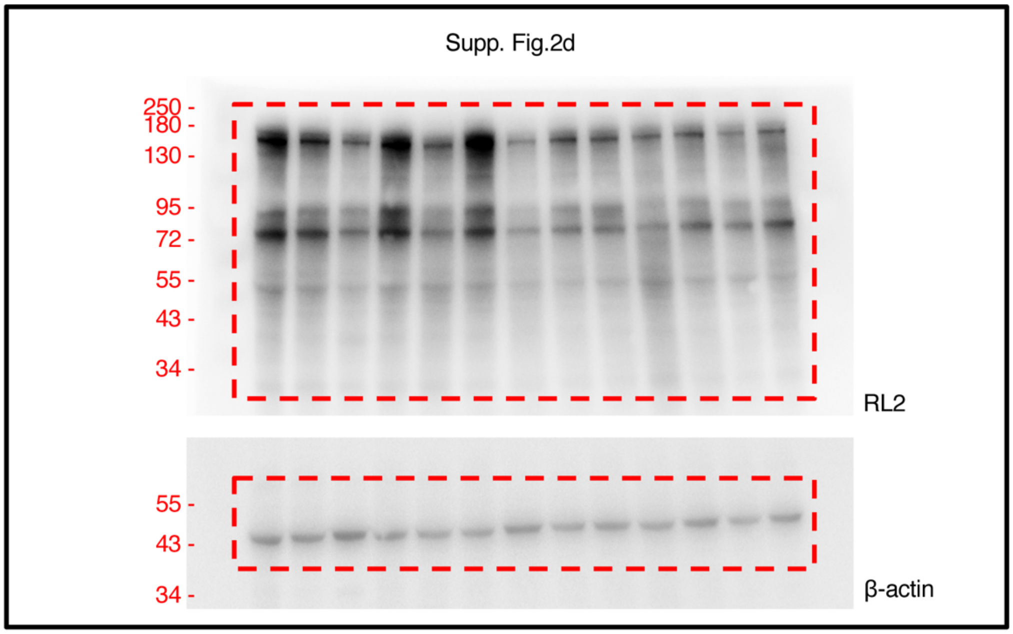

Supplementary Fig. 12. Uncropped blot scans and microscopy images. Part 8.

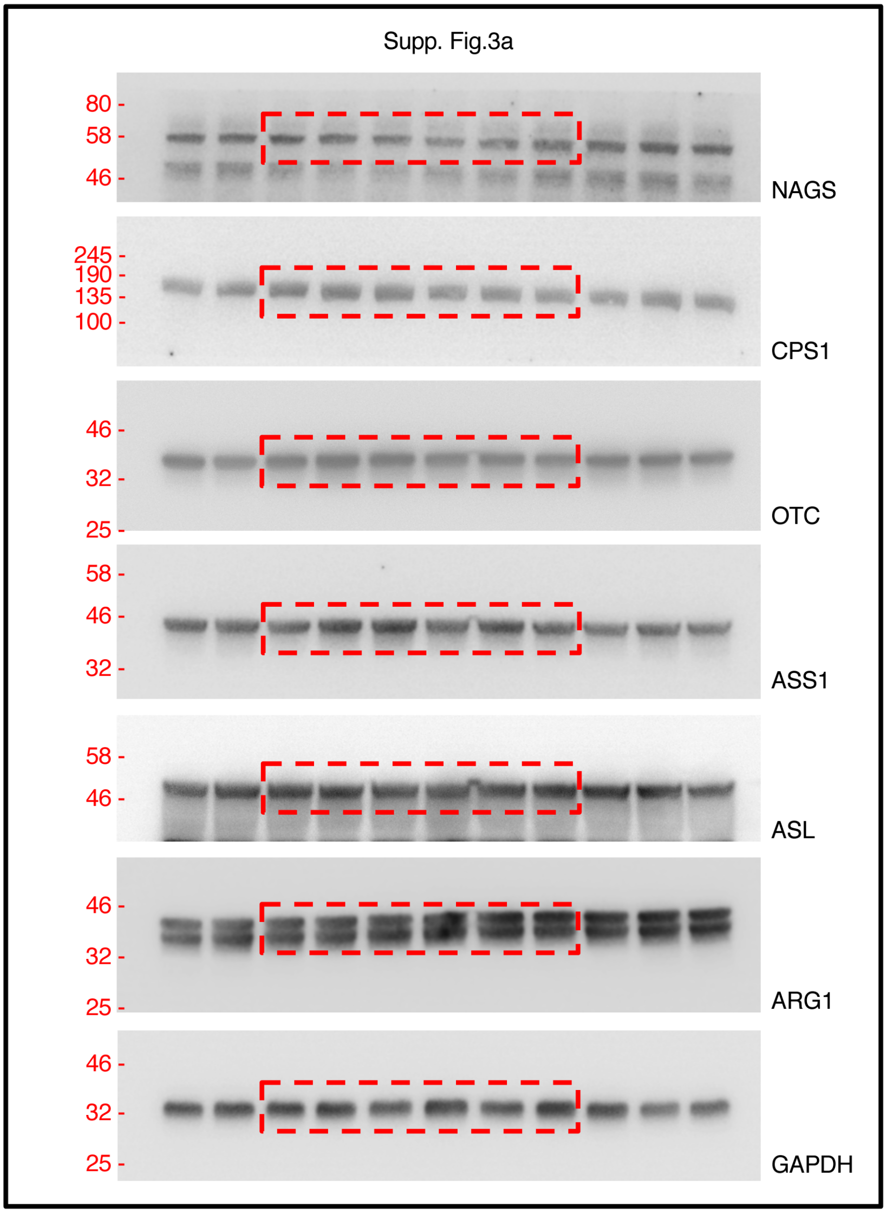

Supplementary Fig. 12. Uncropped blot scans and microscopy images. Part 9.

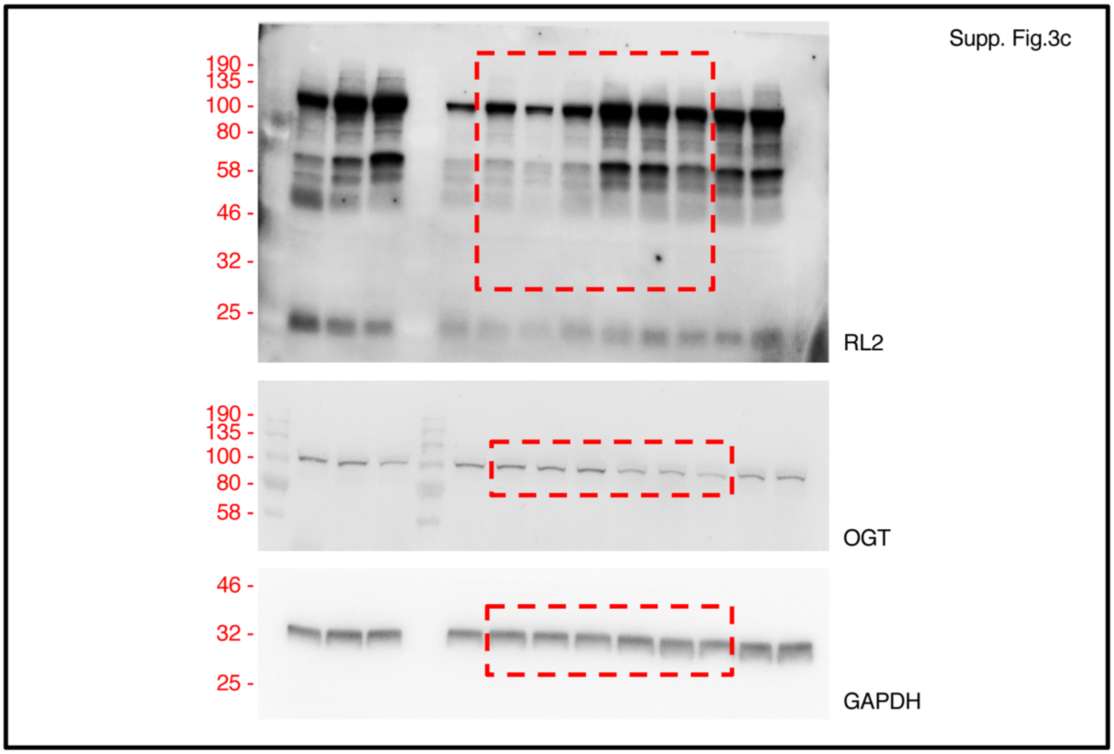

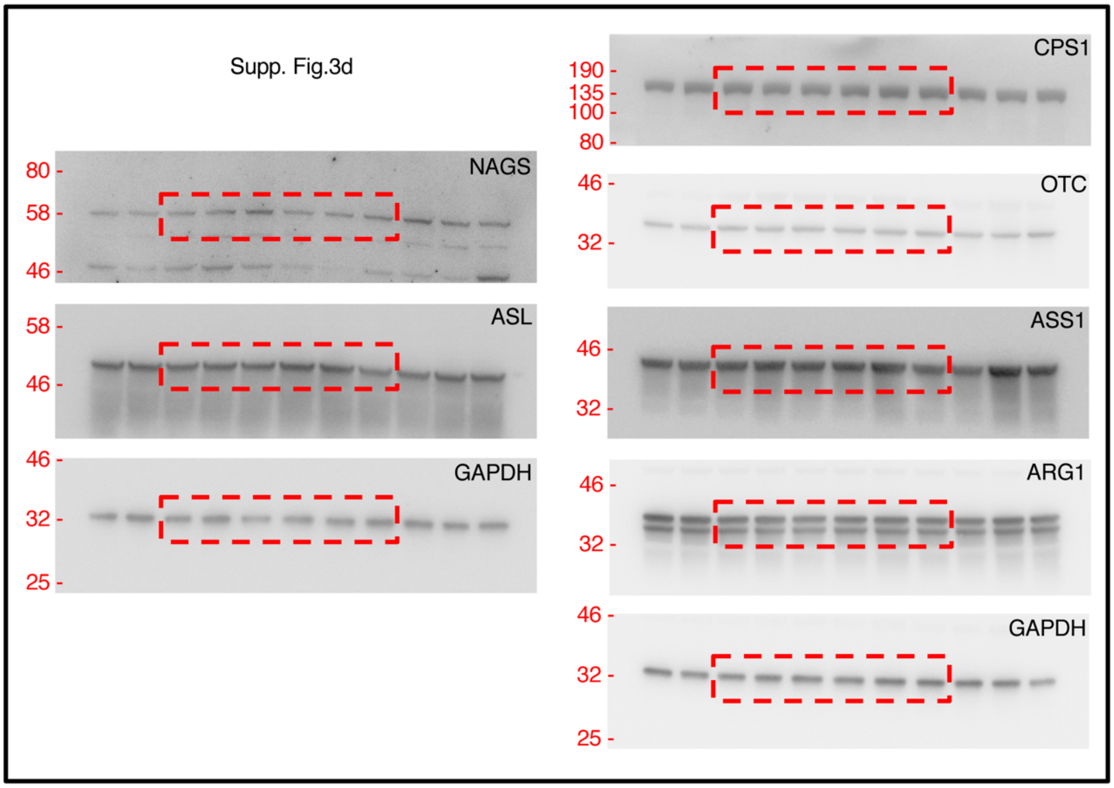

Supplementary Fig. 12. Uncropped blot scans and microscopy images. Part 10.

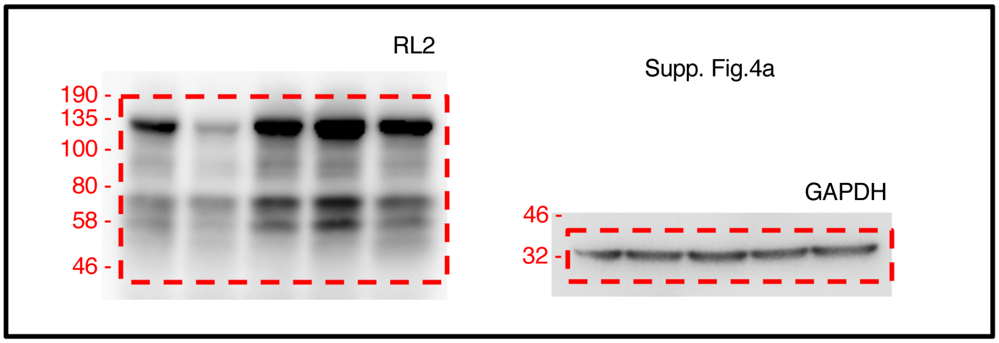

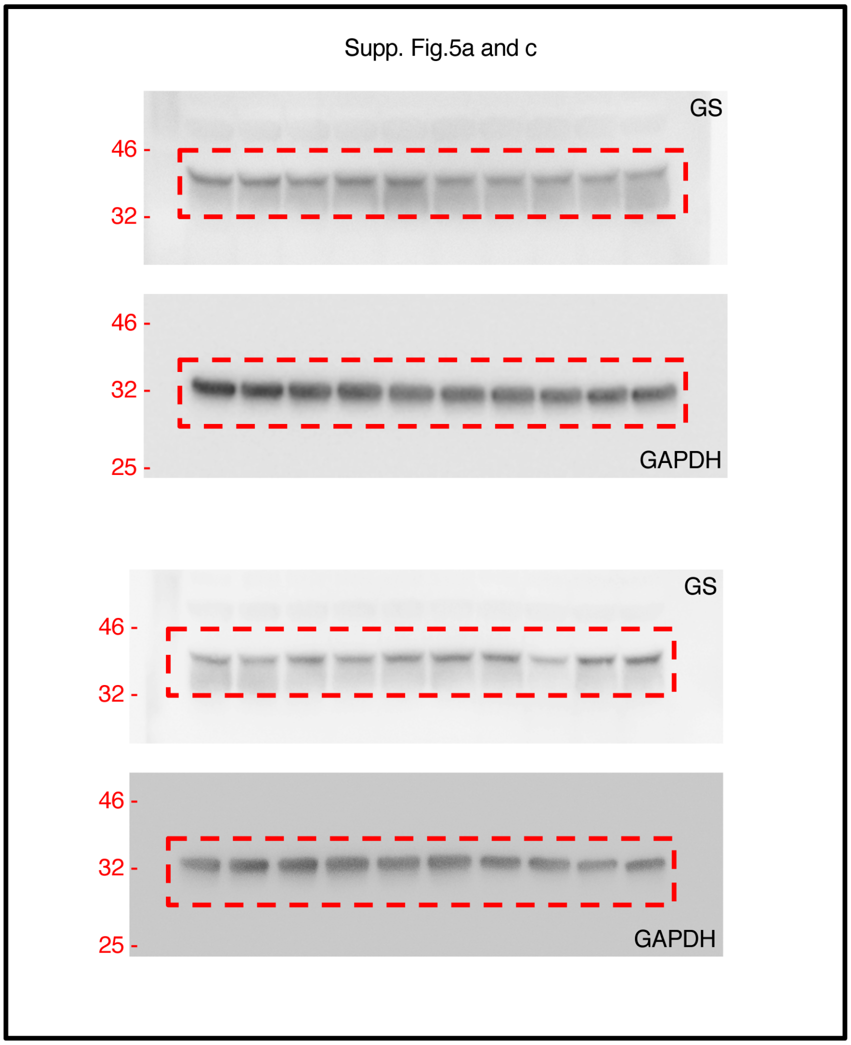

Supplementary Fig. 12. Uncropped blot scans and microscopy images. Part 11.

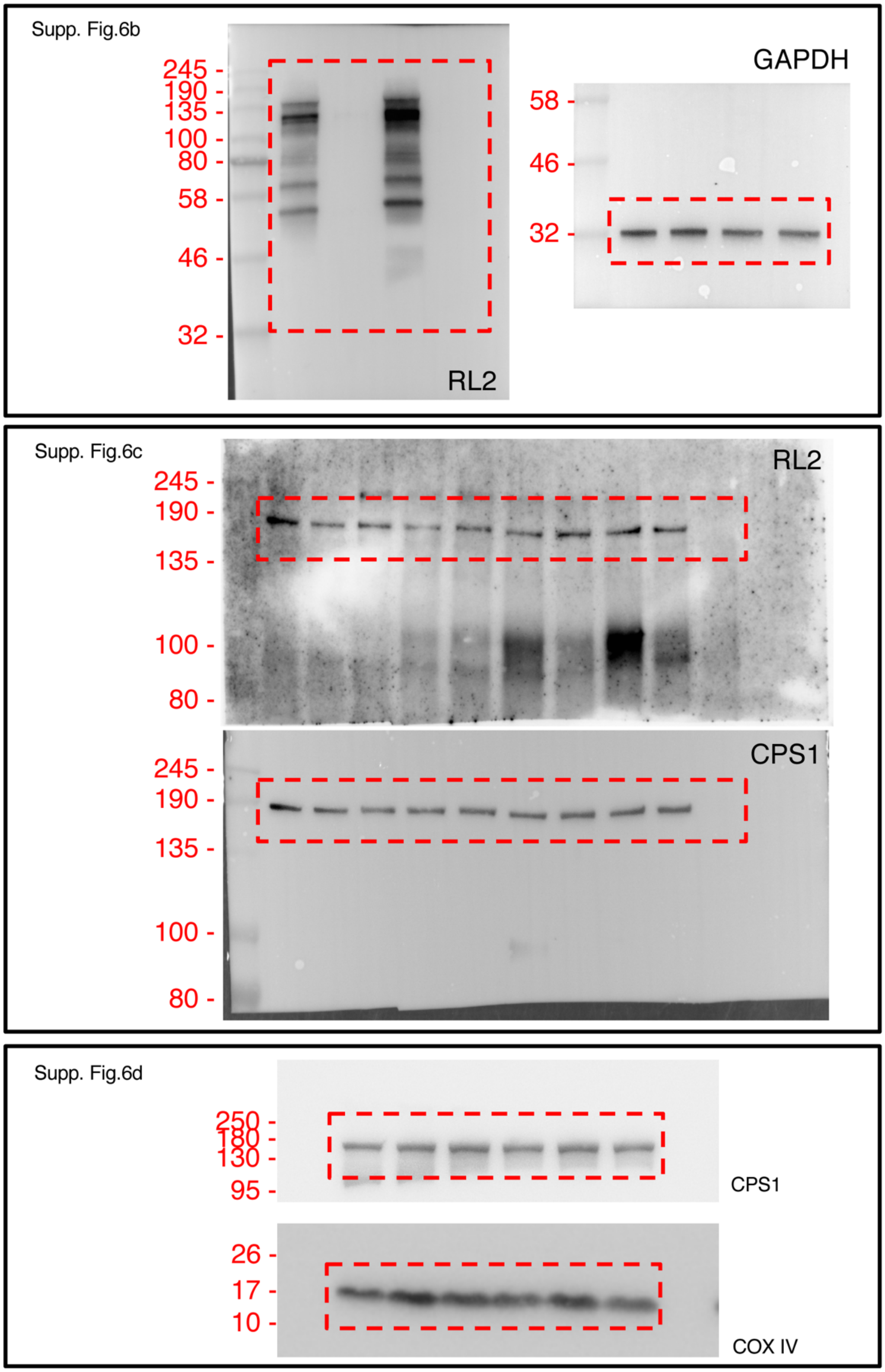

Supplementary Fig. 12. Uncropped blot scans and microscopy images. Part 12.

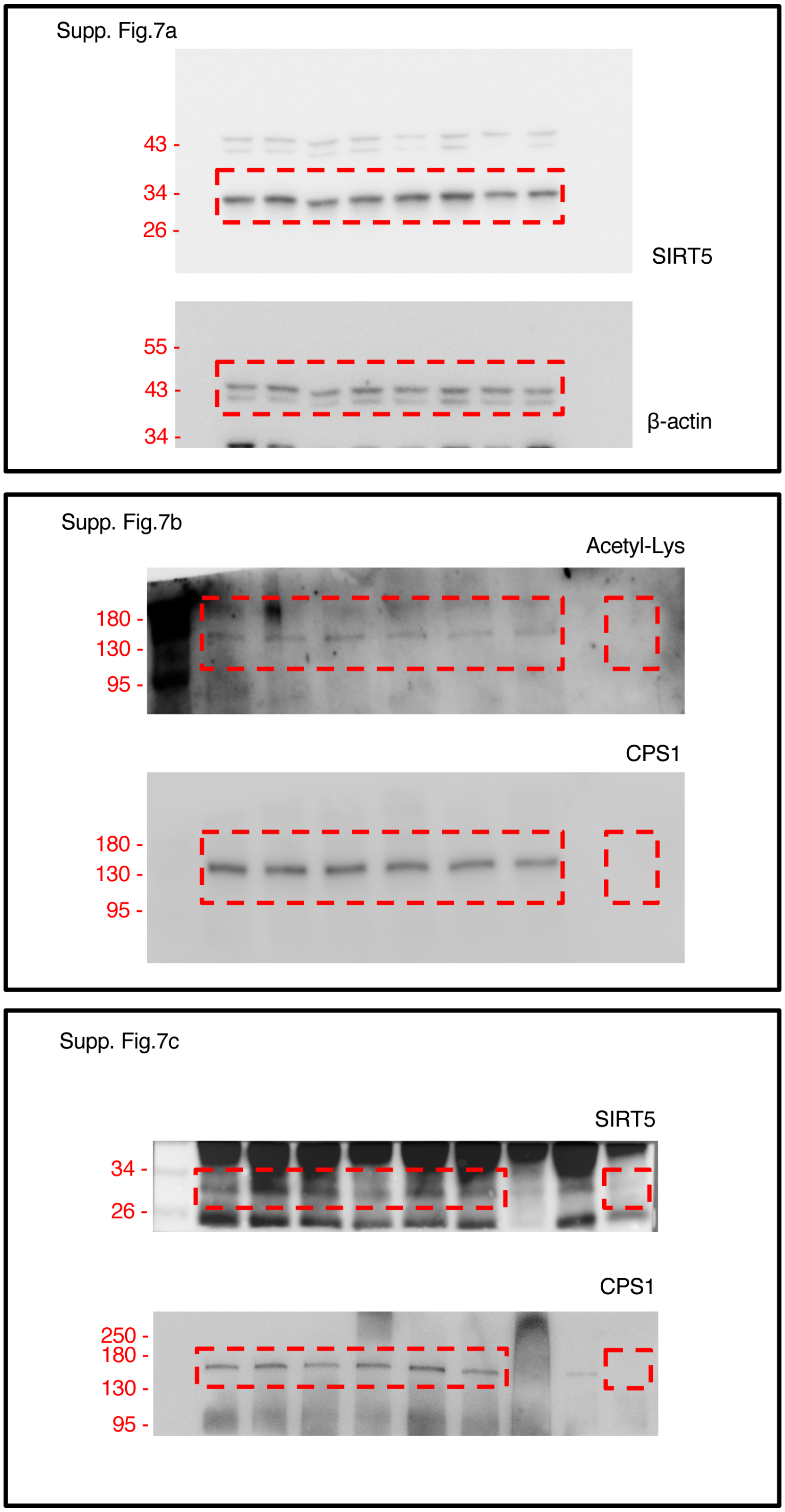

Supplementary Fig. 12. Uncropped blot scans and microscopy images. Part 13.

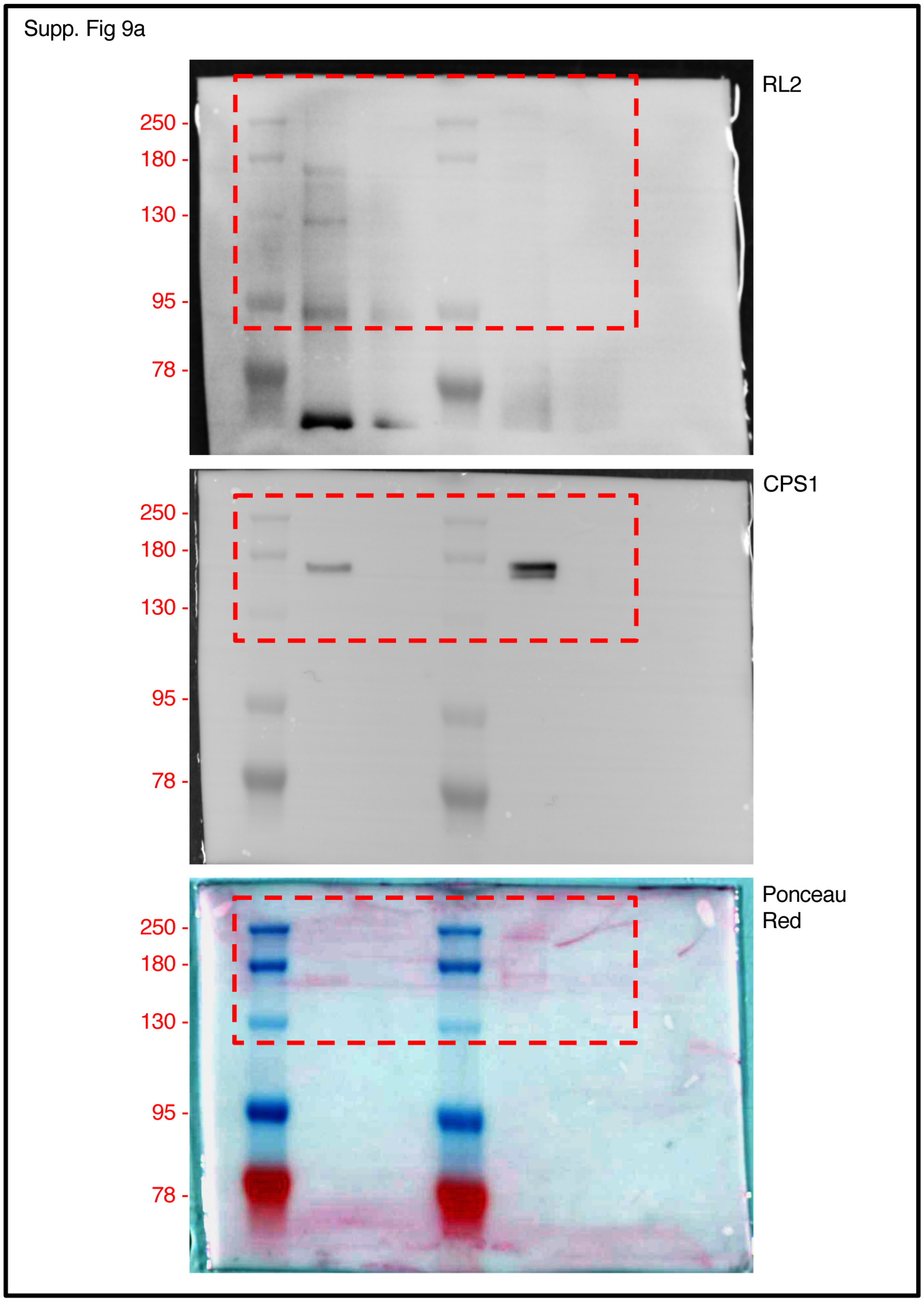

Supplementary Fig. 12. Uncropped blot scans and microscopy images. Part 14.

Supp. Fig 9b

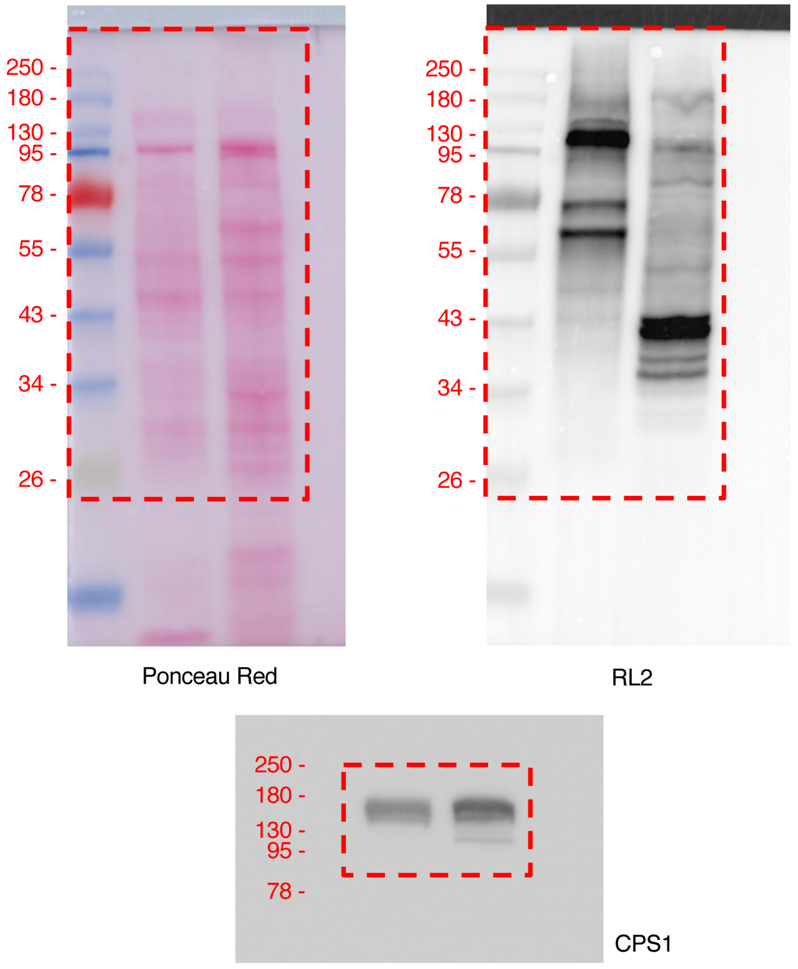

Supp. Fig 9c

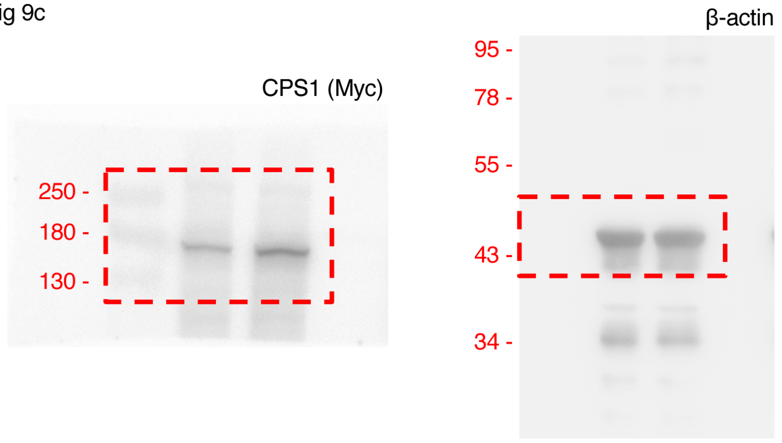

Supplementary Fig. 12. Uncropped blot scans and microscopy images. Part 15.

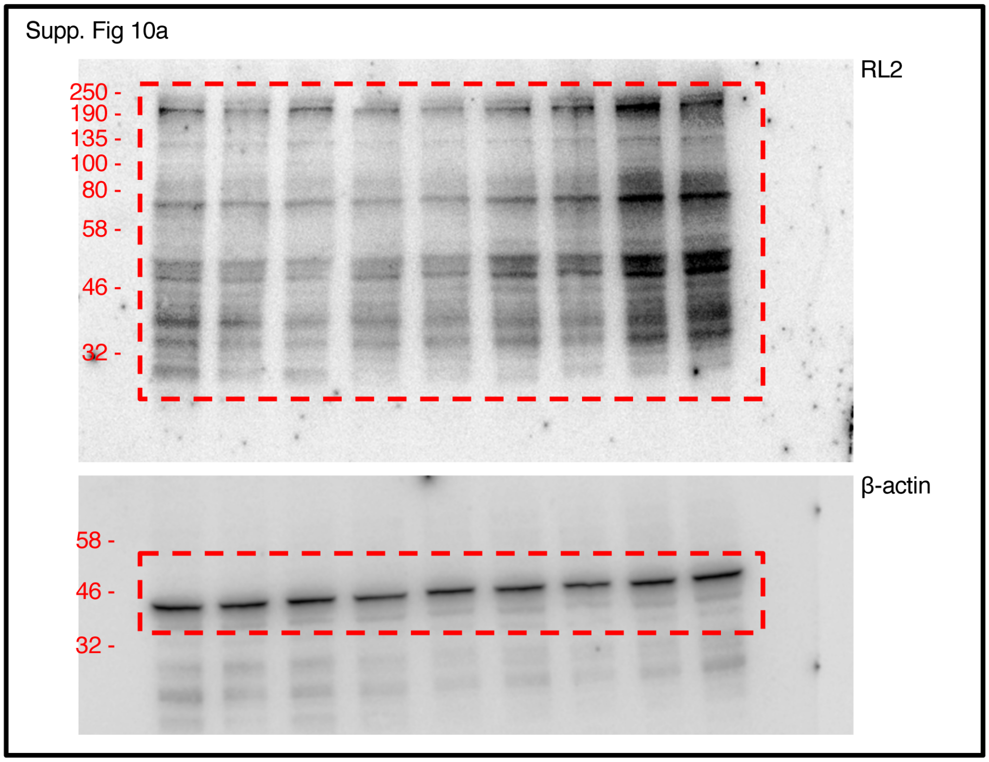

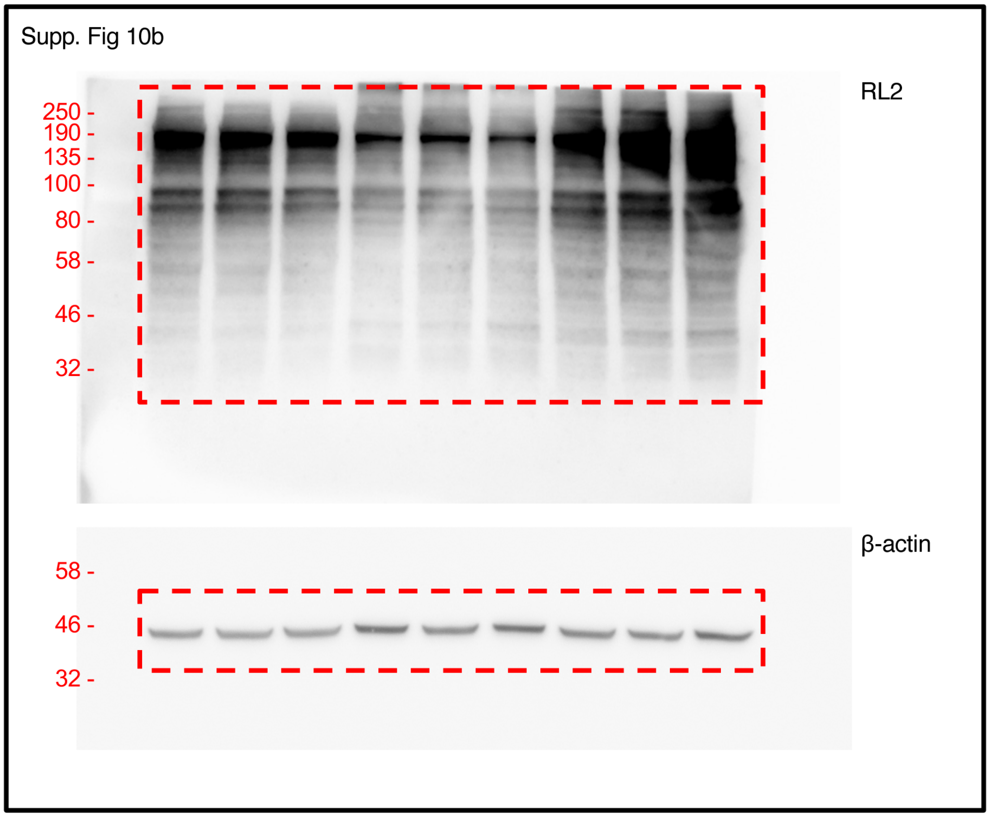

Supplementary Fig. 12. Uncropped blot scans and microscopy images. Part 16.

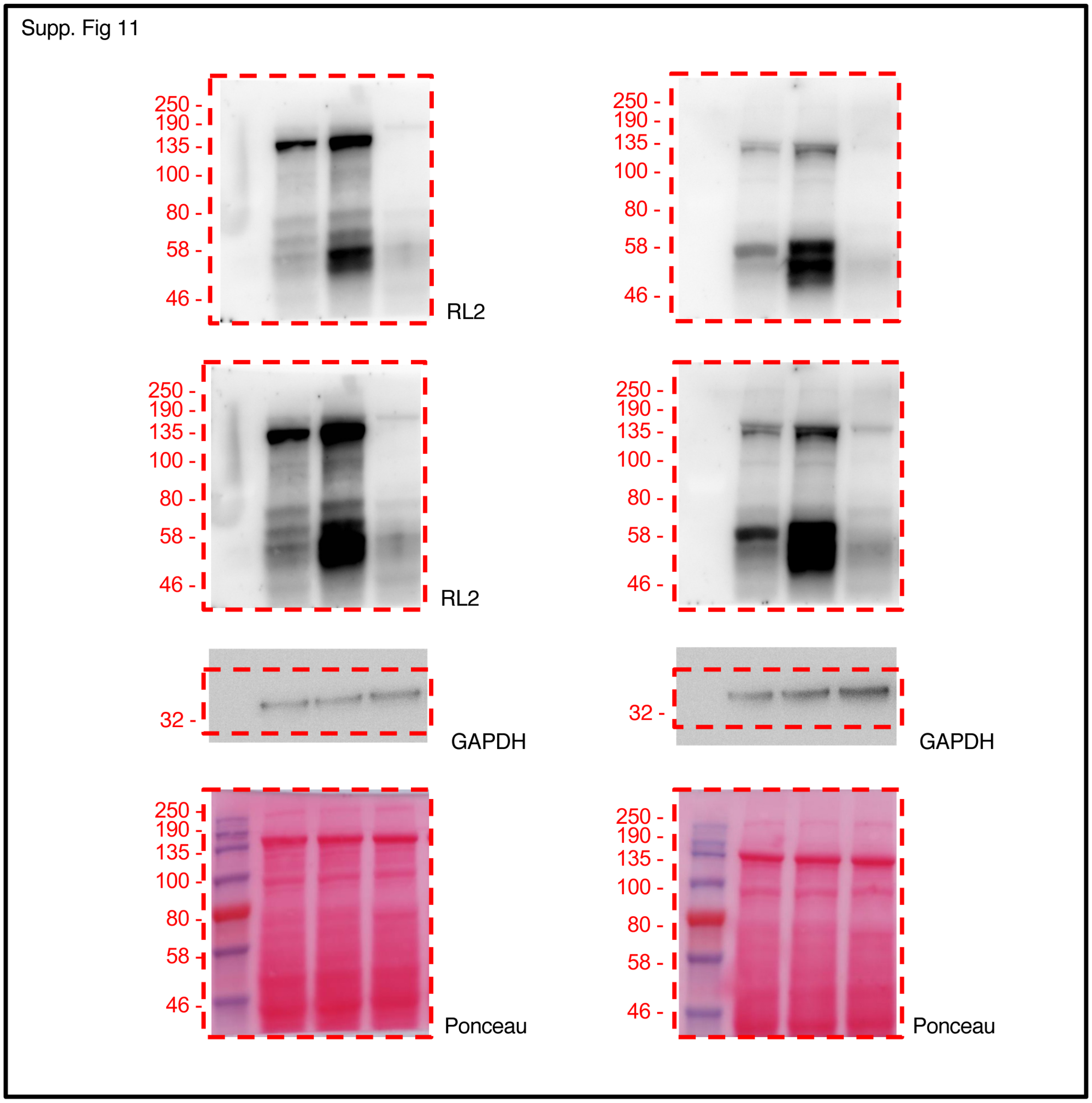

Supplementary Fig. 12. Uncropped blot scans and microscopy images. Part 17.

SUPPLEMENTARY TABLES

Supplementary Table 1. Antibodies used for Western blotting.

Antigen	***Species***	***Source and catalogue number***	***Dilution***
O-GlcNAc (RL2)	Mouse	Abcam, Cat# ab2739	1/ 1,000
O-GlcNAc (CTD110.6)	Mouse	Cell Signaling Technology, Cat #9875,	1/1,000
GFPT1	Rabbit	Proteintech, Cat# 14132-1-AP	1/1,000
OGT	Mouse	Abcam, Cat# ab184198	1/1,000
OGA	Rabbit	Proteintech, Cat# 14711-1-AP	1/1,000
Myc	Rabbit	Cell Signaling Technology, Cat #2278	1/1,000
NAGS	Rabbit	Abcam, Cat# ab65536	1/1,000
CPS1	Rabbit	Abcam, Cat# ab45956	1/1,000
OTC	Rabbit	Novus Biologicals, Cat# NBP1-31582	1/1,000
ASS1	Mouse	Abcam, Cat# ab124465	1/1,000
ASL	Rabbit	Abcam, Cat# ab201026	1/1,000
ARG1	Rabbit	Abcam, Cat# ab91279	1/1,000
Glutamine synthetase	Rabbit	Abcam, Cat# ab16802	1/1,000
SIRT5	Rabbit	Cell Signaling Technology, Cat #8782	1/1,000
Acetylated lysine	Rabbit	Abcam, Cat# ab190479	1/1,000
β-actin	Mouse	Novus Biologicals, Cat# NB600-501	1/3,000
GAPDH	Mouse	Santa Cruz Biotechnology, Cat#sc-32233	1/3,000
Histone H3	Rabbit	Abcam, Cat# ab201456	1/3,000
COX IV	Rabbit	Cell Signaling Technology, Cat # 4844	1/3,000
His Tag	Mouse	Qiagen, Cat# 34660	1/3,000

Supplementary Table 2. Forward (FW) and reverse (REV) primers (5'>3') for mouse and human CPS1 mutagenesis.

Cps1-T109&110 FW: ggggctcccgacgccgctgcccgagacg	*Cps1*-T109&110 REV: Cgtctcgggcagcggcgtcgggagcccc
Cps1-T1078 FW: gtgtcaagatcatgggtgcaagccctctgcagatc	*Cps1*-T1078 REV: Gatctgcagagggcttgcacccatgatcttgacac
CPS1-T109 FW: tgggaatggtggagctcctgatgctactgctctggatg	*CPS1*-T109 REV: catccagagcagtagcatcaggagctccaccattccca
CPS1-T110 FW: tgggaatggtggagctcctgatactgctgctctggatg	*CPS1*-T110 REV: catccagagcagcagtatcaggagctccaccattccca
CPS1-T109&110 FW: tgggaatggtggagctcctgatgctgctgctctggatg	*CPS1*-T109&110 REV: catccagagcagcagcatcaggagctccaccattccca
CPS1-T1078 FW: tcaagatcatgggcgcaagccccctgcag	*CPS1*-T1078 REV: Ctgcagggggcttgcgcccatgatcttga
