# Supplementary material for: O-GlcNAcylation enhances CPS1 catalytic efficiency for ammonia and promotes ureagenesis
Source: Nat Commun. 2022 Sep 5;13:5212. doi: 10.1038/s41467-022-32904-x (PMC9445089; doi:10.1038/s41467-022-32904-x)
Supplement: Supplementary file 2 — Reporting Summary [file 41467_2022_32904_MOESM2_ESM.pdf]

nature portfolio

Corresponding author(s): Nicola Brunetti-Pierri & Leandro R. Soria

Last updated by author(s): Jul 15, 2022

Reporting Summary

Nature Portfolio wishes to improve the reproducibility of the work that we publish. This form provides structure for consistency and transparency in reporting. For further information on Nature Portfolio policies, see our Editorial Policies and the Editorial Policy Checklist.

Statistics

For all statistical analyses, confirm that the following items are present in the figure legend, table legend, main text, or Methods section.

n/a	Confirmed	
☐	☒	The exact sample size (n) for each experimental group/condition, given as a discrete number and unit of measurement
☐	☒	A statement on whether measurements were taken from distinct samples or whether the same sample was measured repeatedly
☐	☒	The statistical test(s) used AND whether they are one- or two-sided<br>*Only common tests should be described solely by name; describe more complex techniques in the Methods section.*
☒	☐	A description of all covariates tested
☐	☒	A description of any assumptions or corrections, such as tests of normality and adjustment for multiple comparisons
☐	☒	A full description of the statistical parameters including central tendency (e.g. means) or other basic estimates (e.g. regression coefficient) AND variation (e.g. standard deviation) or associated estimates of uncertainty (e.g. confidence intervals)
☐	☒	For null hypothesis testing, the test statistic (e.g. F, t, r) with confidence intervals, effect sizes, degrees of freedom and P value noted<br>*Give P values as exact values whenever suitable.*
☒	☐	For Bayesian analysis, information on the choice of priors and Markov chain Monte Carlo settings
☒	☐	For hierarchical and complex designs, identification of the appropriate level for tests and full reporting of outcomes
☒	☐	Estimates of effect sizes (e.g. Cohen's d, Pearson's r), indicating how they were calculated

Our web collection on statistics for biologists contains articles on many of the points above.

Software and code

Policy information about availability of computer code

Data collection	GloMax® Discover Microplate Reader (Promega)<br>Synergy™ NEO Microplate Reader (BioTek)<br>Alliance MINI HD9 AUTO Western Blot Imaging System (UVITEC)<br>Bruker AVANCE III HD-600 spectrometer (Bruker BioSpin)<br>Bruker AVANCE TM III HD-400 (Bruker BioSpin)<br>Mass Spectrometer Polaris Q Trace GC Ultra (Thermo Scientific)<br>Finnigan Delta Plus isotope ratio-mass spectrometer (Thermo Fisher Scientific)<br>Axio Scan Z.1 microscope (Zeiss)<br>Orbitrap Q Exactive HF Mass spectrometer (Thermo Fisher Scientific)<br>Orbitrap Fusion™ Tribrid™ Mass Spectrometer (Thermo Scientific)<br>Xcalibur™ Software (Thermo Scientific)
Data analysis	ImageJ Software (Fiji 2)<br>MaxQuant version 1.5.2.8<br>Proteome Discoverer 2.4.1.15<br>GraphPad Prism 8.0 software

For manuscripts utilizing custom algorithms or software that are central to the research but not yet described in published literature, software must be made available to editors and reviewers. We strongly encourage code deposition in a community repository (e.g. GitHub). See the Nature Portfolio guidelines for submitting code & software for further information.

Data

Policy information about availability of data

All manuscripts must include a data availability statement. This statement should provide the following information, where applicable:

- Accession codes, unique identifiers, or web links for publicly available datasets
- A description of any restrictions on data availability
- For clinical datasets or third party data, please ensure that the statement adheres to our policy

All data generated or analysed included in the main manuscript and its Supplementary Information. Human CPS1 crystal structure (PDB DOI: 10.2210/pdb5DOU/pdb) was used. Source data are provided with this paper. Proteomic data are available via ProteomeXchange with identifier PXD024526.

Field-specific reporting

Please select the one below that is the best fit for your research. If you are not sure, read the appropriate sections before making your selection.

[x] Life sciences [ ] Behavioural & social sciences [ ] Ecological, evolutionary & environmental sciences

For a reference copy of the document with all sections, see nature.com/documents/nr-reporting-summary-flat.pdf

Life sciences study design

All studies must disclose on these points even when the disclosure is negative.

Sample size	Sample size is reported in each figure legend. Sample sizes were based on previous experience (Soria et al. EMBO Mol. Med. 2021, PMID: 33369168; Soria et al. PNAS 2018, PMID: 29279371). A minimum of n=5 per group was included. Sample size was increased if needed to achieve statistical significance. No statistical method was used to determine sample size.
Data exclusions	No data has been excluded for statistical analysis.
Replication	All the experiments were performed at least in triplicates most of them with three or more biological replicates. Further details about the replication have been included in the figure legends.
Randomization	All mice were randomly assigned to experimental groups. In all experiments male mice of the same age were used, except for the experiments with Pcca−/− (A138T) mice, in which both male and females were used. For in vitro experiment, the allocation to the experimental groups was random and the control and treatment wells were plated at the same time.
Blinding	For animal studies, data collection or analysis, investigators were not blinded. Blinding was not deemed to be necessary because the endpoints were not subjective. The laboratory did not had enough funding to support the cost related to the additional unblinded personnel required for the blinding studies.

Reporting for specific materials, systems and methods

We require information from authors about some types of materials, experimental systems and methods used in many studies. Here, indicate whether each material, system or method listed is relevant to your study. If you are not sure if a list item applies to your research, read the appropriate section before selecting a response.

Materials & experimental systems

n/a	Involved in the study	
[ ]	[x]	Antibodies
[ ]	[x]	Eukaryotic cell lines
[x]	[ ]	Palaeontology and archaeology
[ ]	[x]	Animals and other organisms
[x]	[ ]	Human research participants
[x]	[ ]	Clinical data
[x]	[ ]	Dual use research of concern

Methods

n/a	Involved in the study	
[x]	[ ]	ChIP-seq
[x]	[ ]	Flow cytometry
[x]	[ ]	MRI-based neuroimaging

Antibodies

Antibodies used	O-GlcNAc (RL2). Abcam, Cat# ab2739, RRID:AB_303264. Dilution 1/1000 for Western blot (WB) and 1/200 immunohistochemistry (IHC), respectively.<br>O-GlcNAc (CTD110.6). Cell Signaling Technology, Cat #9875, RRID:AB_10950973. Dilution 1/1,000 for WB.

GFPT1. Proteintech, Cat# 14132-1-AP, RRID:AB_2110155. Dilution 1/1,000 for WB.
OGT. Abcam, Cat# ab184198, https://www.abcam.com/ogt--o-linked-n-acetylglucosamine-transferase-antibody-gt678-ab184198.html. Dilution 1/1,000 for WB.
OGA. Proteintech, Cat# 14711-1-AP, RRID:AB_2143063. Dilution 1/1,000 for WB.
Myc. Cell Signaling Technology, Cat #2278, RRID:AB_490778. Dilution 1/1,000 for WB.
NAGS. Abcam, Cat# ab65536, RRID:AB_2149953. Dilution 1/1,000 for WB.
CPS1. Abcam, Cat# ab45956, RRID:AB_941153. Dilution 1/1,000 for WB.
OTC. Novus Biologicals, Cat# NBP1-31582, RRID:AB_2236414. Dilution 1/1,000 for WB.
ASS1. Abcam, Cat# ab124465, RRID:AB_10975633. Dilution 1/1,000 for WB.
ASL. Abcam, Cat# ab201026, https://www.abcam.com/argininosuccinate-lyase-antibody-epr19396-ab201026.html. Dilution 1/1,000 for WB.
ARG1. Abcam, Cat# ab91279, RRID:AB_10674215. Dilution 1/1,000 for WB.
GS. Abcam, Cat# ab16802, RRID:AB_302521. Dilution 1/1,000 for WB.
SIRT5. Cell Signaling Technology, Cat #8782, RRID:AB_2716763. Dilution 1/1,000 for WB.
Acetylated Lys. Abcam, Cat# ab190479, https://www.abcam.com/acetyl-lysine-antibody-rm101-ab190479.html. Dilution 1/1,000 for WB.
Beta-Actin. Novus Biologicals, Cat# NB600-501, RRID:AB_10077656. Dilution 1/3,000 for WB.
GAPDH. Santa Cruz Biotechnology, Cat#sc-32233, RRID:AB_627679. Dilution 1/3,000 for WB.
H3. Abcam, Cat# ab201456, RRID:AB_2650560. Dilution 1/3,000 for WB.
COX IV. Cell Signaling Technology, Cat # 4844, RRID:AB_2085427. Dilution 1/3,000 for WB.
His Tag. Qiagen, Cat # 34660, RRID:AB_2619735. Dilution 1/3,000 for WB.
HRP-conjugated goat anti-mouse. GE Healthcare, Cat #NA931V, RRID:AB_772210. Dilution 1/5,000 for WB.
HRP-conjugated donkey anti-rabbit. GE Healthcare, Cat #NA934V, https://www.cytivalifesciences.com/en/it/search#q=Na934&t=coveo5819fbca. Dilution 1/5,000 for WB.
Anti-mouse IgG isotope. Thermo Fisher Scientific, Cat# 31903, RRID:AB_10959891. Ratio 1 ug/100 ug lysate.

Validation

All antibodies were commercially available and have been validated by companies and/or previous published studies:
O-GlcNAc [RL2]. https://www.abcam.com/o-linked-n-acetylglucosamine-antibody-rl2-ab2739.html. 136 publications. Further validation in Supplementary Fig. 11 of this paper.
O-GlcNAc (CTD110.6). https://www.cellsignal.com/products/primary-antibodies/o-glcnac-ctd110-6-mouse-mab/9875. 56 publications. Further validation in Supplementary Fig. 11 of this paper.
GFPT1. https://www.ptglab.com/products/GFPT1-Antibody-14132-1-AP.htm. 17 publications.
OGT. https://www.abcam.com/ogt--o-linked-n-acetylglucosamine-transferase-antibody-gt678-ab184198.html. 5 publications.
OGA. https://www.ptglab.com/products/MGEA5-Antibody-14711-1-AP.htm. 41 publications.
Myc. https://www.cellsignal.com/products/primary-antibodies/myc-tag-71d10-rabbit-mab/2278. 522 publications.
NAGS. https://antibodyregistry.org/search.php?q=AB_2149953. 2 publications.
CPS1. https://www.abcam.com/cps1-antibody-ab45956.html. 16 publications.
OTC. https://www.novusbio.com/products/ornithine-carbamoyltransferase-antibody_nbp1-31582. 3 publications.
ASS1. https://www.abcam.com/ass1-antibody-2b10-ab124465.html. 8 publications.
ASL. https://www.abcam.com/argininosuccinate-lyase-antibody-epr19396-ab201026.html. 3 publications.
ARG1. https://www.abcam.com/liver-arginase-antibody-ab91279.html. 55 publications.
GS. https://www.abcam.com/glutamine-synthetase-antibody-ab16802.html. 32 publications.
SIRT5. https://www.cellsignal.com/products/primary-antibodies/sirt5-d8c3-rabbit-mab/8782. 44 publications.
Acetylated Lys. https://www.abcam.com/acetyl-lysine-antibody-rm101-ab190479.html. 12 publications.
Beta Actin. https://www.novusbio.com/products/beta-actin-antibody-ac-15_nb600-501. 500 publications.
GAPDH. https://www.scbt.com/it/p/gapdh-antibody-6c5. 3904 publications.
H3. https://www.abcam.com/histone-h3-antibody-epr17785-ab201456.html. 17 publications.
COX IV. https://www.cellsignal.com/products/primary-antibodies/cox-iv-antibody/4844. 291 publications.
His Tag. https://www.qiagen.com/us/products/discovery-and-translational-research/protein-purification/tagged-protein-expression-purification-detection/anti-his-antibodies-bsa-free/. 42 publications.
HRP-conjugated goat anti-mouse. http://antibodyregistry.org/AB_772210. 290 publications.
HRP-conjugated donkey anti-rabbit. https://www.cytivalifesciences.com/en/it/search#q=Na934&t=coveo5819fbca. 331 publications.
Anti-mouse IgG isotope. https://www.thermofisher.com/antibody/product/Mouse-IgG-Isotype-Control/31903. 44 publications.

Eukaryotic cell lines

Policy information about cell lines

Cell line source(s)	Primary human hepatocytes (Supplier: Gibco™. Source donor: Single Donor). Human Huh-7 Hepatic Cell Line (Supplier: JCRB Cell Bank - JCRB0403).
Authentication	For human hepatocytes: expression of CPS1 protein. For Huh 7 cells morphology was checked by microscope.
Mycoplasma contamination	Primary hepatocytes and Huh 7 cells used in the study were not tested for mycoplasma.
Commonly misidentified lines (See ICLAC register)	No commonly misidentified cell lines were used.

Animals and other organisms

Policy information about studies involving animals; ARRIVE guidelines recommended for reporting animal research

Laboratory animals	Male 6-week-old C57BL/6 wild-type mice were purchased from Charles River Laboratories, Calco, Italy. Four-week-old male and female Hypomorphic Pcca−/− (A138T) mice (from Michael Barry, PhD Lab at Mayo Clinic, Rochester, Minnesota, USA) were maintained on a C57BL/6 background. Details of housing, husbandry conditions, gender and age were also included in the text (Methods) and in the corresponding figure legends.
Wild animals	The study did not involve wild animals.
Field-collected samples	No field collected samples were used in this study.
Ethics oversight	All mouse procedures were performed in accordance with regulations and were authorized by the Italian Ministry of Health; the National Institutes of Health and the Institutional Animal Care and Use Committee of David Geffen School of Medicine at UCLA (Los Angeles, United States); and the Spanish Law of Animal Protection.

Note that full information on the approval of the study protocol must also be provided in the manuscript.
