# Supplementary material for: O-GlcNAcylation enhances CPS1 catalytic efficiency for ammonia and promotes ureagenesis
Source: Nat Commun. 2022 Sep 5;13:5212. doi: 10.1038/s41467-022-32904-x (PMC9445089; doi:10.1038/s41467-022-32904-x)
Supplement: Supplementary file 3 — Description of Additional Supplementary Files [file 41467_2022_32904_MOESM3_ESM.pdf]

Title: Supplementary Data 1
Description: Liver O-GlcNAcylated proteins identified by MS.

Title: Supplementary Data 2
Description: CPS1 site mapping.
